# Statistical Modeling for the Prediction of Infectious Disease Dissemination With Special Reference to COVID-19 Spread

**DOI:** 10.3389/fpubh.2021.645405

**Published:** 2021-06-16

**Authors:** Subhash Kumar Yadav, Yusuf Akhter

**Affiliations:** ^1^Department of Statistics, School of Physical and Decision Sciences, Babasaheb Bhimrao Ambedkar University, Lucknow, India; ^2^Department of Biotechnology, School of Life Sciences, Babasaheb Bhimrao Ambedkar University, Lucknow, India

**Keywords:** distribution fitting models, time series regression models, epidemiological models of disease, parameters, estimation, prediction

## Abstract

In this review, we have discussed the different statistical modeling and prediction techniques for various infectious diseases including the recent pandemic of COVID-19. The distribution fitting, time series modeling along with predictive monitoring approaches, and epidemiological modeling are illustrated. When the epidemiology data is sufficient to fit with the required sample size, the normal distribution in general or other theoretical distributions are fitted and the best-fitted distribution is chosen for the prediction of the spread of the disease. The infectious diseases develop over time and we have data on the single variable that is the number of infections that happened, therefore, time series models are fitted and the prediction is done based on the best-fitted model. Monitoring approaches may also be applied to time series models which could estimate the parameters more precisely. In epidemiological modeling, more biological parameters are incorporated in the models and the forecasting of the disease spread is carried out. We came up with, how to improve the existing modeling methods, the use of fuzzy variables, and detection of fraud in the available data. Ultimately, we have reviewed the results of recent statistical modeling efforts to predict the course of COVID-19 spread.

## Introduction

Statistical modeling and prediction in epidemiology provide a method to understand why and how infections spread and how they might be prevented or restricted. For instance, when a new infectious disease emerges or there is an outbreak of a known infectious disease, epidemiologists are the scientists, who collect, analyze, and interpret information to indicate interventions for halting further dissemination. Many infectious diseases do not respect national boundaries, color, creed, caste, communities, etc. initially affecting only one region of the world, and rapidly disseminates to other regions and ultimately may become a pandemic like COVID-19 (1). These diseases may have several types based on their extent of dissemination.

*EPIDEMIC*

A disease that influences enormous people within a community, population, or region may be called an epidemic.

*PANDEMIC*

An epidemic that develops in many countries or continents is known as a pandemic.

*ENDEMIC*

A disease that is related to a specific group of people or a country is known as endemic.

*OUTBREAK*

When an epidemic is disseminating to a larger extent than its expected number of cases, it is called an outbreak. It may be one case in a new locality. If, it is not registered soon, there may be an outbreak causing the epidemic.

The advent of commercial airlines globally has ensured that the time between the appearance of a new pathogen and its worldwide spread is much more sooner than ever before. Outbreaks of viral hemorrhagic fevers (VHFs) like Ebola and Lassa fever, and other respiratory viruses, such as influenza or SARS-Cov2, typically attracted the attention of media and politicians due to the potentially high rate of infectivity and mortality. An infectious disease epidemiologist is concerned with a range of pathogens—from viruses, bacteria, and fungi to the infesting eukaryotic worms. Infectious agents are also an important cause of subsequent disease e.g., some infections, such as hepatitis C virus (HCV) and human papillomavirus (HPV), and bacteria like *Helicobacter pylori*, etc. cause cancers. Moreover, the statistical and empirical analysis in the discipline of epidemiology allows us to take information from individuals and to aggregate it into logical groups (defined by the characteristics of the person, the environment, or time points). It may help us to logically understand from where the infection has originated, how it might be disseminating, and thereby the potential means for prevention and its containment in the restricted zones. This kind of theoretical analysis of the data collected from the field may be useful for the generation of hypotheses. To formally test a hypothesis that aims to explain these observations, epidemiologists require more sophisticated approaches that use a variety of study designs to minimize bias and statistical methods to quantify the role of chance (2). The aims of such analysis may be:

1. To understand the extent of any of the infectious diseases in a given population in terms of transmission, new cases, and existing cases;
2. To analyze the prognosis and natural history of infections, including links to diseases not previously considered to be of infectious origin;
3. To determine the infection causing a particular disease and the risk factors that increase the frequency of infection acquisition and progression from infection to disease, sequelae, and different clinical outcomes;
4. To assess the efficacy and effectiveness of preventive and curative measures;
5. To inform the policymakers that would help in prevention, control, and eventual elimination of the infectious disease.

An understanding of the host response to microbial pathogen exposure is a prerequisite for studying infectious diseases (1). Similarly, the development of preventive and post-infection therapeutic interventions such as vaccines, antibiotics, and antiviral agents continues to play a central role in epidemiology and control of infectious diseases:

1. To develop vaccines, which train our immune system by pre-exposure of attenuated infectious agents or subunits of such agents, before the real exposure of the microbial pathogen of the particular infectious diseases. Vaccines have remained central to protect and control infectious diseases.
2. To discover novel antibiotics as the discovery of antibiotics has changed the complete history of bacterial infections and their epidemiology. Regrettably, antimicrobial resistance emerged almost immediately and has now reached alarming levels globally.
3. Although antivirals, historically have had modest effectiveness, recent advances have been made in the treatment of diseases caused by viral pathogens like the human immunodeficiency virus (HIV) and hepatitis C.

Infectious disease epidemiology has also been influenced by the emergence of new ideas and the technological advent of biomedical sciences (system scale “omics” based technologies such genomics, transcriptomics, proteomics, and metabolomics), as well as advances in disciplines, such as immunology, cell biology, and microbiology, which have expanded our horizons about the biology and epidemiology of infections as well as human opportunities for therapeutic interventions. For instance, stratified therapy, where the choice of treatment regimen takes into account the genetic make-up of the host as well as the microbial pathogen. It has become possible with advancements in faster and cheaper genomic sequencing technologies. Nonetheless, this has been accompanied by added complexity in terms of analyzing the “big data” generated and the tools required to undertake such analyses, given the mammoth volume of the data generated (3). There are several major constituent factors of infectious disease epidemiology that are important to study with their detailed intricacies; we are highlighting them in brief here.

### The Trinity of the Microbial Pathogen, the Host, and the Environment

The dissemination kinetics of infections must be taken into consideration when investigating the spread of infections and it is further required to design presumptive measures required for the disease control. Three major elements attribute the overall outcome in this context that influences each other in a very complex way and contribute to the dissemination of infectious disease.

It is an exclusive feature of any of the infectious diseases, that their causative microbial agents may be only transmitted from a person to another person (or from animals to the people, or from animals to animals), leading to sustained spread and may ultimately leading to an outbreak that may require prompt public health action. Another unique characteristic of infections is that some animals, typically insects, may serve as vectors to transmit the infection to humans. Examples include mosquitoes that can carry the dengue virus or the parasite that causes malaria and the Triatominae that carry the parasite that causes Chagas' disease (4).

Transmission in such ways may show recurrent patterns frequently observed with infectious diseases, which may vary in their predictability. It is logical to imagine that, such a pattern may be described by a simple mathematical equation. If essential determinants of the observed pattern can be described, such a model might provide a mechanism to assess the impact of interventions and to inform the planning of disease control. The contagious nature of infections also means that, for most infectious diseases, the impact of any single case and its public health and economic consequences may go beyond those attributable to the loss of quality of life and risk of death to that individual. Cancer or other non-infectious metabolic diseases may result in a substantial loss of healthy life-years among those directly affected; however, a single infection could spread and eventually affect many of the individuals in a population. Therefore, evaluation of the public health impact of interventions against infections is usually the use of models that account for the non-static nature of infectious disease dissemination.

Another unique feature of infectious diseases is the observation and quantification of individuals that carry subclinical infections, therefore, they are asymptomatic carriers or who are in the preclinical or convalescent phase of illness may still transmit the infection to others. In the case of some of the infectious diseases, such as tuberculosis (caused by the bacterium, *Mycobacterium tuberculosis*), AIDS (caused by virus HIV), hepatitis (caused by hepatitis virus), etc., the microbial pathogen may remain in dormant or latent inside the host organism's body, such cases also remain undetectable (5). Therefore, this implied that knowledge of the observed number of symptomatic cases of a particular infection alone may not be sufficient to fully understand the trends or to evaluate the effects of therapeutic interventions applied.

The outcome of any of the infectious diseases also depends upon the natural history of an infection in the human host by its previous levels of exposure to the microorganism or by active or passive immunization. For some infectious diseases, immunity can be conferred for life, while repeat episodes due to recurrence or reinfection are possible for other infections. The genomes of microbial pathogen and the host determine the behavior, response to the drug administered, pathogenicity, and virulence of the pathogen, and thus modulate the outcome of the disease to varying degrees; the human genome also influences the susceptibility of the host, as well as its response to the microbial infection as well as to the therapeutic interventions such as drugs and the vaccines. Although classical genetics methods allowed the identification of some drug resistance mutations and typing of strains, next-generation sequencing (NGS) methodologies have made possible the analysis of the entire genes of pathogens, leading to zoom out understanding of their evolution (phylogenetics), transmission patterns, and the drug resistance (4).

Different types of microbes and human hosts interact *via* complex mechanisms, in different parts of the host body, such as the gut, where an abundant set of usually risk-free and advantageous bacteria resides and called the gut microbiome may alter the behavior of pathogenic microbes when they enter into the gut. The relatively new discipline of metagenomics aims to analyze the full genomic material of hosts and the coexisting microbes both the symbionts and the pathogens (6). Once a person is exposed to a microbial pathogen, they may become infected. The time between exposure and the onset of the symptoms of the disease is referred to as the incubation period. The average period between two equivalent stages of consecutive infected cases is the serial interval, which is most frequently measured at symptom onset, due to the clinical ease of defining this time point. Moreover, some of the individuals are asymptomatic and yet disseminate the organisms to other individuals, which could be called a carrier state. Herd immunity refers to the indirect protection of individuals in a population who are not vaccinated or otherwise immune; this indirect protection arises as a consequence of the immunity of others in their community (7).

The sources of infections include symptomatic infected humans, animals, and the environment. For instance, respiratory viruses such as COVID19 may be spread by coughing and aerosolization of the microbe that is then inhaled, causing infections in susceptible people. Some of the infectious diseases, such as typhoid fever, may be transmitted by asymptomatic human carriers, while others are acquired from animals (e.g., zoonotic infections such as salmonella) or the environment (e.g., Legionnaires' disease). These infectious diseases may be disseminated directly from one infected individual to another e.g., sexually (HIV), by touching (scabies), by biting (rabies), or vertically from mother to child (rubella and cytomegalovirus (CMV) or indirectly *via* a vector or vehicle (food- and water-borne pathogens, healthcare-associated infections (HAIs), e.g., an infected catheter). Infections can also be spread by droplets over very short distances (Ebola) and by droplet nuclei, which are smaller and can travel longer distances (airborne transmission, e.g., influenza and tuberculosis (TB) (8).

### The Host Response

Ultimately, the immune response from the host is the most important key factor which determines the outcome of any of the microbial infections. Generally, this pertains to both parts of the host's immune system, the innate (from the hereditary) and the adaptive (acquired) immunity. The innate immune system includes physical barriers like the skin and diverse secretions of the body, as well as cells of the immune system like mast cells, histiocytes, macrophages, dendritic cells, Kupffer cells, and Langerhans cells. These cells have surface molecules known as receptors, which may be able to differentiate between molecules that belong to our bodies and coming from outside of the body. These cells take part in the initiation of the inflammation that may attract the white blood cells and other phagocytes (cells that are capable of ingesting and destroying the pathogens). Their competence may vary with the age and physiological status of the host. The innate system is cosmopolitan in nature and present in all organisms, but the adaptive immune system is developed in later time points of evolution along the tree of life, this has first appeared in vertebrates. It is responsible for immune memory and this helps the organisms to respond to specific pathogens, that they may have encountered previously. The principle of vaccines relies on such memory. In general, antigens are identified by specific white blood cells known as lymphocytes. As explained above, adaptive immunity may be acquired through previous exposure to the pathogen, as well as through active and passive immunization (4). The implication of host immunity on the worldwide burden of infections is remarkable. Various studies of the global burden of the disease generate “big data” from a diverse source to highlight the changing patterns of infectious diseases and the efficacy of therapeutic interventions such as drugs and vaccines in use. Remarkable declines in mortality from many infections have been observed after implementation of the public immunization and other therapeutic programs against many infectious diseases such as diarrheal diseases, polio, lower respiratory infections, TB, and measles (9).

Traditional approaches to manage and control infections have not been effective in tackling the spread of many microbial pathogens. Currently, these approaches are being extended with the addition of novel technologies, however, the classical principle of infectious disease control remains conserved. Behind any efficient infectious disease control program requires a robust surveillance system. A strategic plan to control infectious disease must rely on sound knowledge of infectious disease epidemiology, evidence-based public health analysis of the data leading to effective policymaking and implementation, and optimal communication between all of the stakeholders.

The progressing evolution and emergence of novel germs, such as Severe acute respiratory syndrome-coronavirus2 (SARS-Cov2), SARS-Cov, Middle East Respiratory Syndrome coronavirus (MERS-Cov), and new forms of known pathogens, such as Ebola virus and Avian influenza, and changing dissemination patterns of such infections, such as Dengue fever (DF) and Chikungunya, re-emergence of previously endemic infections, and the development of antimicrobial resistance, such as methicillin-resistant *Staphylococcus aureus* (MRSA), all these pose a serious threat to the control of infections For the foreseeable future, infections are likely to remain a challenge due to the movement of pathogenic species between animals and humans and the ability of microorganisms to evolve *via* mutations and the exchange of plasmids. The importance of infectious disease epidemiology is, therefore, here, to stay in the future as well. It is well-known that the preparation of a vaccine for any infectious disease is a very lengthy process even it takes a longer time to get the medicine for the same. As these diseases spread at reasonably high rates and are dangerous for human lives, therefore, till the medicine or the vaccine is not prepared, prevention is better than cure policy is at the disposal. It is of paramount importance to know with what rate it is infecting the people, what is the death rate, what is the recovery rate, how many hospital beds are required, and how many people are going to be affected by any particular infectious disease, therefore, it may be controlled. This information is of crucial importance in making the best policies to prevent the disease. To know all such types of information, statistical modeling plays a very important role. There are mainly three methods to model the spread of these infectious diseases including the current pandemic of COVID19. In this review, we attempt to discuss these three methods namely, distribution fitting, time series regression models, and epidemiological modeling in detail.

## The Distribution Fitting

**Figure 1:**
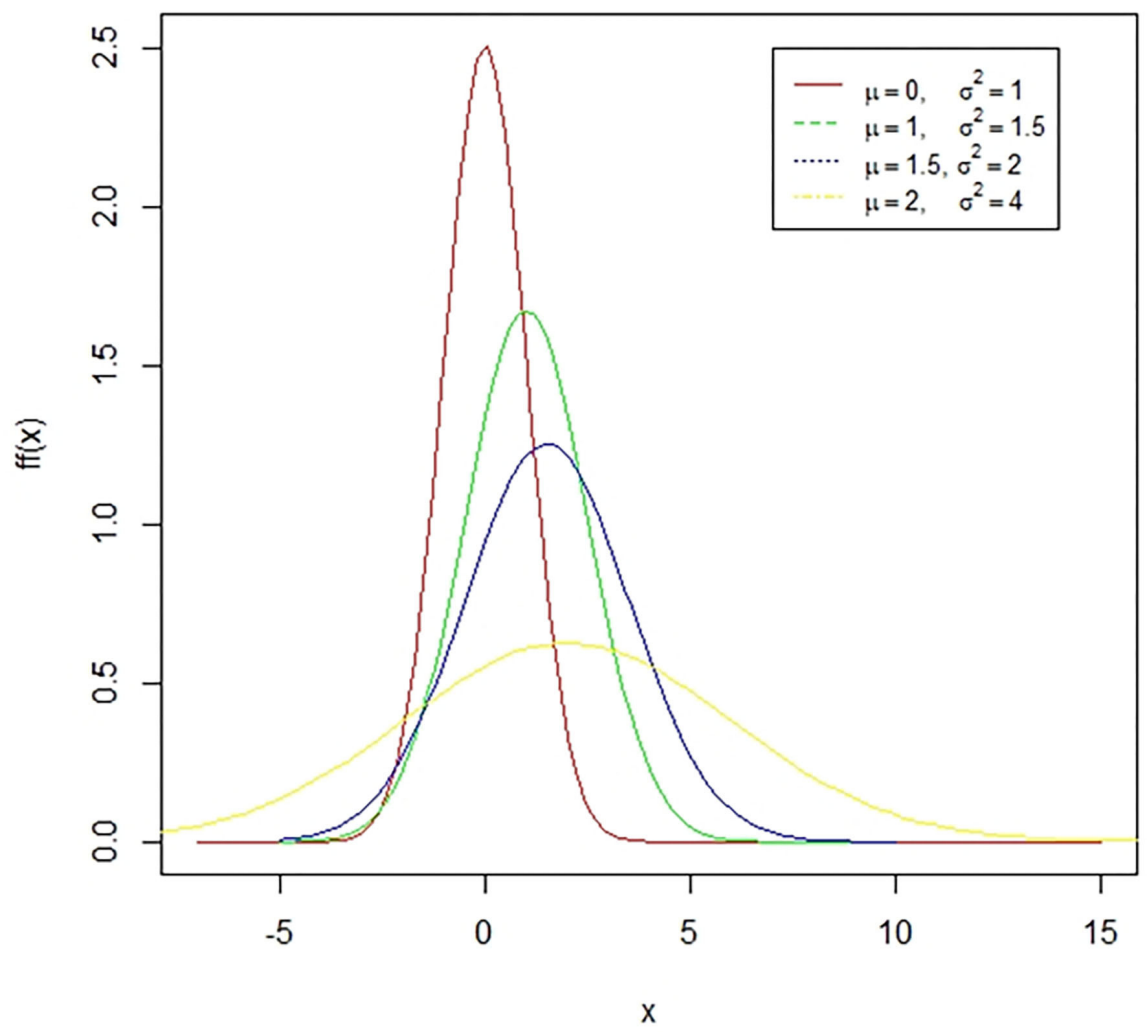
A case of normal distribution. The normal distribution is a symmetric bell-shaped curve. The standard normal distribution has a mean zero and a standard deviation of one. The coefficient of skewness for this distribution is zero while the coefficient of kurtosis is three. The presented figure represents different normal curves for different values of means and variances.

**Figure 2:**
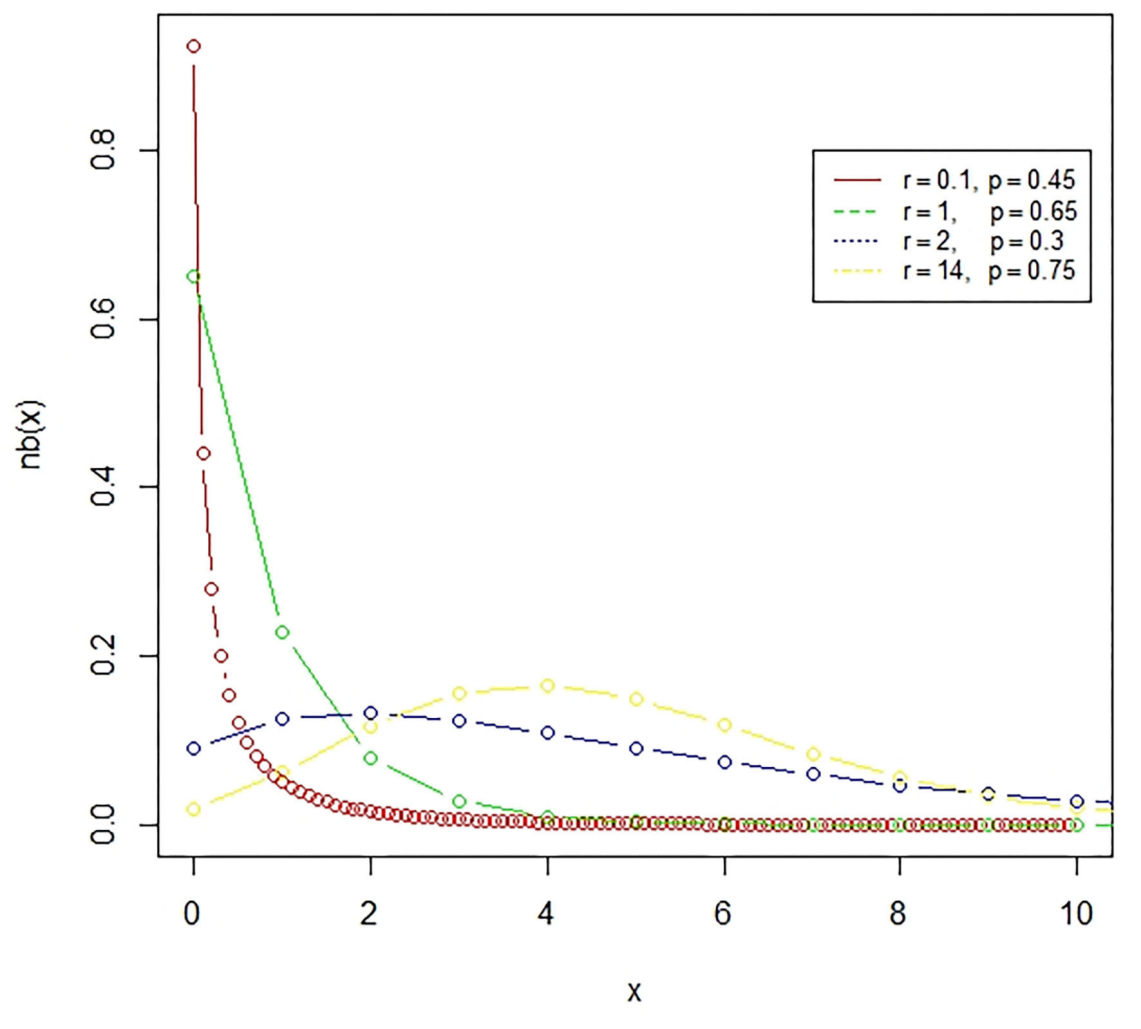
The negative binomial distribution is a discrete probability distribution, which represents the number of failures in a sequence of independent and identically distributed Bernoulli trials till the required number of successes occurred.

**Figure 3:**
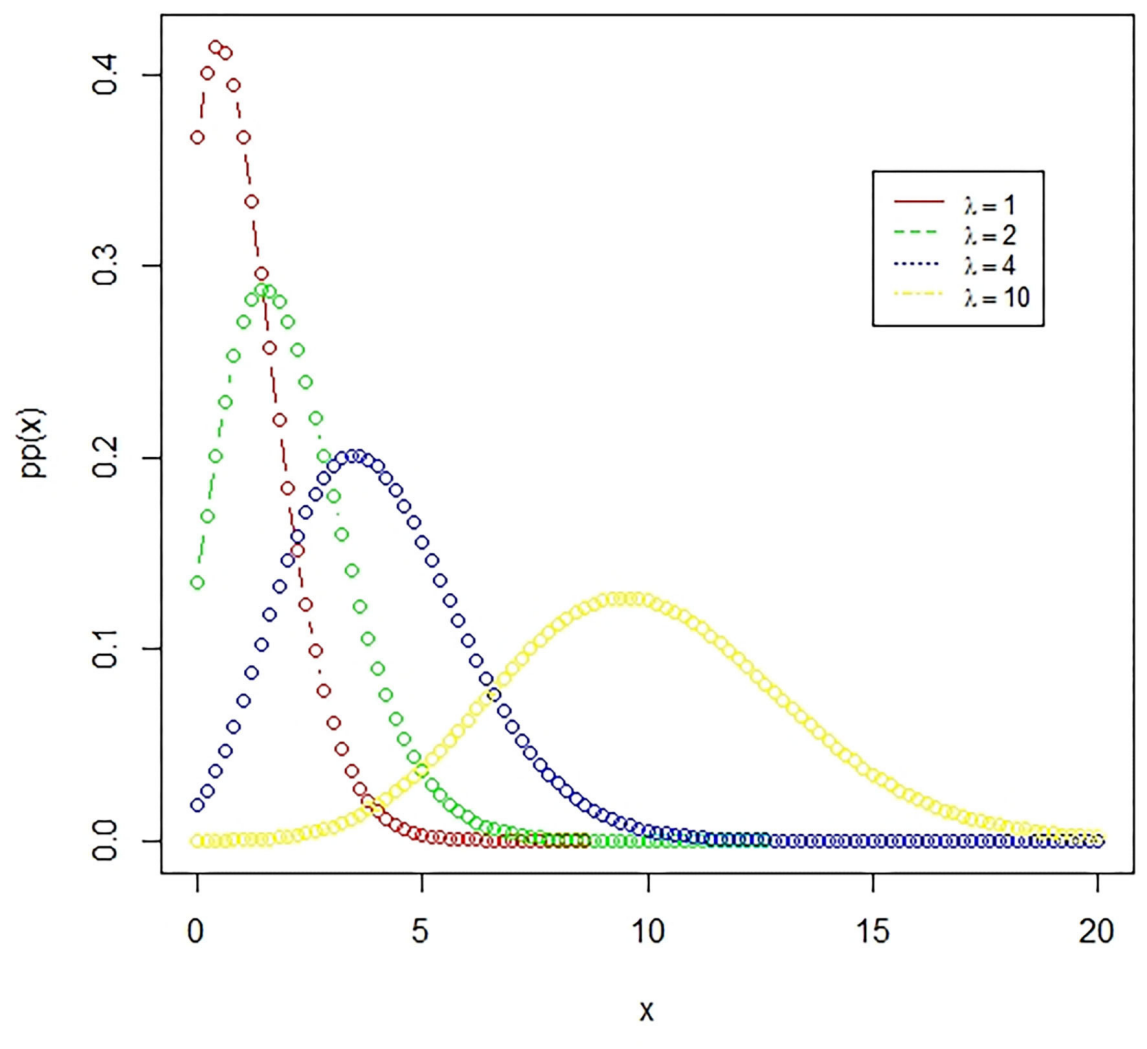
The Poisson distribution. A Poisson distribution represents the probability of happening of rare events, where the number of favorable outcomes is very low in comparison to the total events. The presented figure represents different Poisson curves for different values of the parameter.

**Figure 4:**
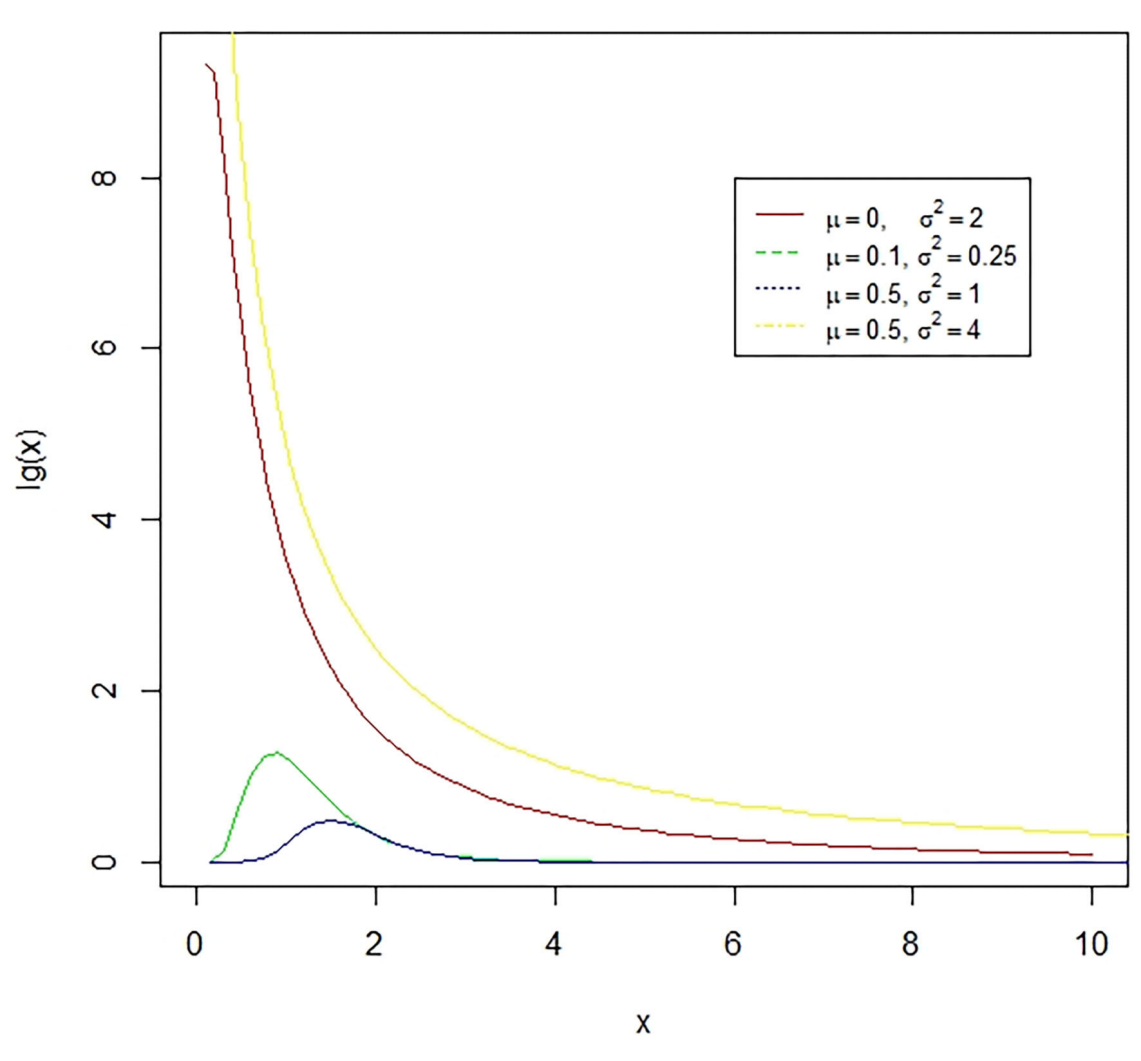
The lognormal distribution is a continuous distribution of a random variable whose logarithm has a normal distribution. E.g., if X is log-normally distributed, then *Y* = *log*(*X*) will have a normal distribution. The presented figure represents different normal curves for different values of means and variances.

**Figure 5:**
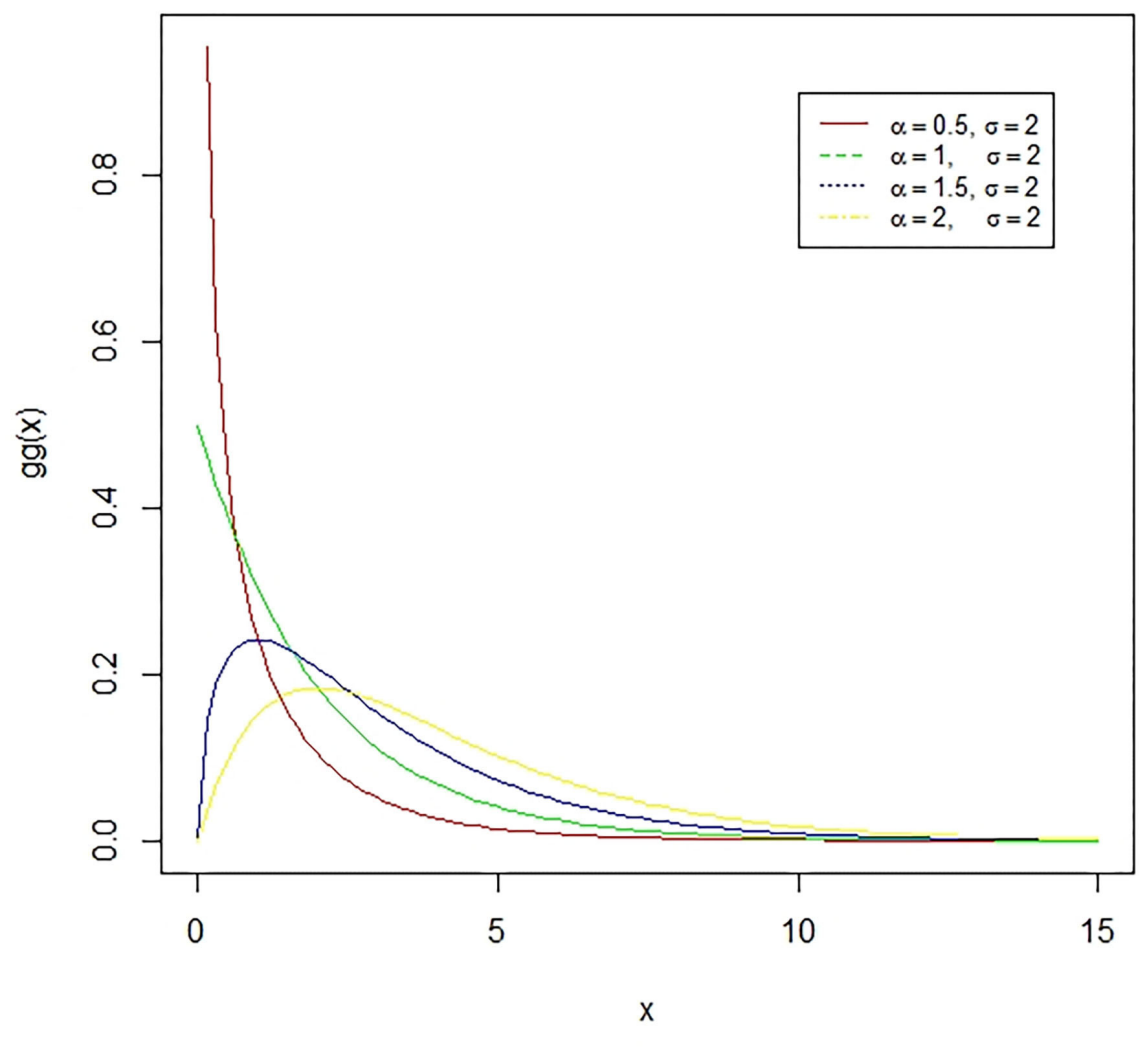
The Gamma distribution is the distribution of a random variable X for which *E*(*X*) = κθ = $\frac{\alpha }{\sigma }$ is fixed and greater than zero, and *E*[*log*(*X*)=ψ(κ) + log(κ) = *log*(α) − *log*(σ) is fixed (ψ is the digamma function). The presented figure represents different gamma curves for different values of the parameters α and σ.

Since infectious diseases spread over time and the behavior and kinetics of infection dissemination depends on the stages of the epidemic, the mean number of infections by one infected person and the time in which the persons show symptoms, etc., thus, it is the growth rate of infection which determines the total number of infections. It depends on the numerous factors such as health infrastructure and testing protocols, total population, literacy, environmental conditions of the country, complexity, heterogeneity, and dynamism of the human behaviors, government interventions, etc. (10). It has been observed that the epidemic spreads in different stages, for instance, COVID-19 in most of the countries shown to be in six stages. The growth rates for different countries are different at different stages. The stages have been defined by the structural breakpoints on the time series data. Some researchers have fitted different theoretical distributions like Normal, Negative Binomial, Poisson, Gamma, Exponential, and Lognormal, etc. (Figures 1–5) for different stages or the break interval on the availability of the data for the total infected days of the particular stage (11). Datta et al. (11) fitted different distributions for different stages and shown that in most of the stages Lognormal distribution is the best-fitted distribution among the class of discrete as well as continuous distributions for COVID-19 worldwide data. They have used Chow F-test statistic to determine different potential breakpoints for COVID-19 data series that is they have used the Chow test to determine potential breakpoints for different stages and to test the consistency of the parameters' estimates for the fitted distributions for each stage. The breakpoints are decided for the fitted distribution on the least values of the fitting measures like residual sum of squares (RSS), Akaike information criteria (AIC), Bayesian information criteria (BIC), mean absolute error (MAE), etc. (12). As it is well-established that the infectious disease mainly depends on two factors, the number of carriers and the time of infections, therefore Datta et al. (11) have found the structural breaks for each country under consideration for the total cumulative cases of COVID-19 for different stages. They have defined five breaks for different countries and fitted different distributions and shown Lognormal to be the best-fitted one for almost all stages and all countries. They have also talked about the average number of days for different stages and other measures of the distribution along with the P-P and Q-Q plots to see the fitting of the distributions for different stages. Following are the generally fitted figures for different distributions and the overall graph of the countries which have overcome the disease COVID-19.

Many more researchers have worked in the field, which along with their unique contributions are presented in the following Table 1.

**Table 1:** Fitting of different distributions for different infectious diseases.

**S. no**.	**References**	**Conclusion drawn**
1	Meyer and Held (62)	The authors have studied the short-time human travel behavior through power Law (Pareto, Uniform, Cauchy, etc.) concerning the distance. They used extended space-time models for influenza infectious disease surveillance data to better capture the dynamics of disease spread. They have studied the statistical properties of the best-fitted distribution for a better explanation and prediction of influenza.
2	Virlogeux et al. (63)	In this work a novel avian influenza virus, influenza A(H7N9) emerged in China was studied. The authors have fitted different parametric and non-parametric distribution for A(H7N9) incubation periods and studied the properties of the fitted distributions. The best fitted parametric distribution observed was Weibull distribution and the mean incubation period was 3.4 days with a 95% confidence interval [3.0 3.7] and the variance was 2.9 days. The results were very similar for the non-parametric Turnbull estimate as well.
3	Virlogeux et al. (64)	The authors studied Middle East Respiratory Syndrome coronavirus (MERS) disease in the Arabian Peninsula and in South Korea in 2015. They examined the incubation period distribution of MERS coronavirus infection using parametric (Lognormal, Gamma, Weibull, Exponential, Log-logistic) and non-parametric (turnbull) methods. They have shown that Gamma and Weibull are best-fitted distributions for South Korea while Lognormal and Log-logistic are the best fitted for Saudi Arabia and estimated a mean incubation period of 6.9 days with 95% credibility interval as [6.3 7.5] for cases in South Korea and 5.0 days with 95% credibility interval as [4.0 6.6] among cases in Saudi Arabia.
4	Hanel et al. (65)	The authors worked on the most standard methods based on maximum likelihood (ML) estimates of power-law function which is an exponential distribution. The best-fitted power function distribution based on the fitting measures was observed after that the appropriate ML estimator was derived for arbitrary exponents of power-law distributions on bounded discrete sample spaces. They had shown that a similar estimator was also working for continuous data. This ML estimator was implemented and its performance was compared with previous works. Further, a general protocol was given on how it could be used for estimating the spread of the infections.
5	Li et al. (66)	In this study, prediction and parameter estimation of infections were studied using noisy case reporting data. A simple stochastic, discrete-time, discrete-state epidemic model was established with both process and observation errors and was used to characterize the efficiency of different flavors of Bayesian Markov chain Monte Carlo (MCMC). They fitted different parametric distributions with ceilings (binomial and beta-binomial distributions) and without ceilings (Poisson and negative binomial) and the best-fitted distribution were studied for the statistical properties to explain and prediction of the nature of the infections.
6	De-Souza et al. (67)	The authors inferred that climate change has a high impact on governing the health and death rates due to respiratory system diseases and remained poorly understood by probability distribution modeling. They fitted the Burr, Inverse Gaussian, Lognormal, Pert, Rayleigh, and Weibull distributions to respiratory diseases, and the shape and scale parameters of the distributions were determined to verify the quality of fit through fitting measures. The lognormal and Rayleigh are best observed fit for hospital admissions.
7	Valvo (68)	The author studied the epidemiological model for the prediction of the time trends of COVID-19 deaths worldwide. They have taken a bimodal distribution function as a mixture of two lognormal distributions to model the time distribution of deaths in a country. They mentioned that an asymmetric lognormal distribution is better fitted in comparison to symmetric distribution functions. Based on the best model, they have further analyzed and predicted the future behavior of the spread of COVID-19 and was extrapolated until the end of the year 2020.
8	Vazquez (69)	The author has shown that infection spreads are expected to grow exponentially in time but their initial kinetics is not well understood. In this study, derivation of the analytical expressions was carried out for the kinetic behavior with a gamma distribution of generation intervals. Omitting the exponential distribution, the spread of the infection grows as a power law at short times. At long times, the kinetics is exponential with a growth rate estimated by the reproductive number and the parameters of the generation interval distribution. These kinetic derivations can be deployed to do better estimates of parameters used for infection spread.
9	El-Monsef (70)	The author has fitted finite mixture of m-Erlang distributions to analyze the COVID-19 dissemination. The author has derived different moments and shape parameters estimate for the suggested model and shown that it has a bound hazard function. A special case of the suggested distribution has also been discussed along with the predictive technique to estimate the parameters of the fitted distribution. In this fitted distribution, the data of the COVID-19 cases from Egypt was used to examine the flexibility of the proposed model.
10	Almetwally et al. (71)	The authors suggested a model for fitting the COVID 19 mortality rates in the UK and Canada using optimal statistical technique. They have suggested a new two-parameter lifetime distribution by combining inverted Topp-Leone (ITL) and modified Kies inverted Topp-Leone (MKITL) distributions. They have shown that the suggested model has various important properties as simple linear representation, hazard rate function, and moment function. They have used various methods of estimation for the estimation of parameters of the suggested distribution. They have shown through the data simulation study on COVID-19 cases that the suggested model is better than the traditional methods.
11	Mubarak and Almetwally (72)	The authors have introduced a new extended three-parameter exponential distribution and studied the survival function and hazard function. They have also used the maximum likelihood estimation (MLE) and maximum product spacing (MPS) methods for to evaluate the parameters of this distribution. An empirical study is carried out to judge the superiority of the suggested model over some well-known distributions using COVID-19 data and it was concluded that the suggested distribution is better fitted over competing distributions.
12	Gonçalves et al. (73)	In the presented work, authors have concluded that the inaccurate epidemiological concepts are being used during COVID-19 pandemic. They pointed out about social media and scientific journals regarding wrong references for “normal epidemic curve” and “log-normal curve/distribution” and the textbooks and courses of reputed institutions have spread slightly incorrect information. Most of them have shown histogram as epidemic curve or using epidemic data as Gaussian distribution, ignoring its property of temporal indexing. The authors have further observed that epidemic curve may be of Gaussian curve type and be modeled from Gauss function but it could not be a perfect normal distribution or a log-normal, as some of the previous studies have shown. Further, they have mentioned that a pandemic gives highly-complex data and to handle it effectively, there is need to go beyond the “one-size-fits-all solution” of statistical and mathematical modeling. Finally they suggested that the classical textbooks should be updated on pandemics and epidemiology should give reliable information to policy making and implementation.

In our opinion, since the infectious disease spreads over time so there must be only one continuous graph or the distribution which should be followed by the complete data like the S-shaped curves introduced by Malthus (13) and not in different stages like Datta et al. (11) has proposed. As the behavior of the curve is changing in different stages and different distributions are best fitted for different stages, so it is not justified to present a single problem in different stages while it must be presented through a single distribution to present the real picture so that actual policies may be made. As it is a big data problem so there must be a normal distribution as the best one to define the infectious diseases. One more drawback with the stages distribution fitting is the required data as the required sample size may not be obtained due to the unavailability of the data so the estimates may not as good as they should be. As far as the Chow test is concerned regarding the determination of the breakpoints and the consistency checking of the estimates of parameters of the fitted distribution, it may be significant as there are rapid changes in different stages even within the stage as may be seen from the graph given by Datta et al. (11).

## Time Series Regression Modeling

Time series regression modeling and forecasting of infectious diseases are of paramount importance for knowing the behavior of the disease spread and to make better policies to overcome the problem. The prime purpose of time-series regression modeling is to gather the past information very carefully and rigorously on a scale of time for the construction of the most suitable model that may appropriately explain the natural framework of the series. Then the constructed model is applied for forecasting the future values of the series. Thus, the forecasting through the constructed time series model may be considered as an act through which the future is predicted by the past (14). As the epidemic develops over time, so it is important to study its trending behavior know many things like when it is going to finish, when it will have a peak, and how many persons will be affected by it. Many active researchers have worked and working on infectious disease modeling for several years. Since the time series is crucial importance in disease modeling, it is to be mentioned that due attention must be kept fitting an appropriate model for the said time series. Naturally, the best time series prediction relies on the most suitable fitted model.

Various researchers have developed the time series models over many years to enhance the prediction precision of the disease. Several crucial models have been suggested by various authors for enhancing the accuracy as well as the efficiency of time series models and their forecasting as well. The main time series models used for forecasting the infectious diseases are auto regressive time series models like AR (Auto Regressive), MA (Moving Average), ARMA (Auto Regressive Moving Average), ARIMA (Auto Regressive Integrated Moving Average), and SARIMA (Seasonal Auto Regressive Integrated Moving Average). The following Table 2 developed from Zhang et al. (15), represents different autoregressive models in two categories, stationary and non-stationary time series models.

**Table 2:** Stationary and non-stationary time series regression models used in epidemiology.

**S. no**.	**Time series model**	**Model description**
**Stationary time series regression model**		
1.	Autoregressive model (AR)	Present values explicated linearly based on previous values and present residuals
2.	Moving Average (MA)	Present values of time series explicated linearly for previous values and the time series residuals
3.	Autoregressive Moving Average (ARMA)	As a combination of AR and MA, present values of time series explicated linearly for current values but also previous and present residuals
**Non-stationary time series regression model**		
4.	Autoregressive Integrated Moving Average (ARIMA)	Based on the ARMA model, but a differencing procedure transforming non-stationary data to stationary data
5.	Seasonal Autoregressive Integrated Moving Average (SARIMA)	Based on the ARIMA model, but also includes seasonal differencing, in case of data has periodic patterns

These time series models help forecast the forthcoming propensity of the phenomenon, risks, and distribution or dilation trend of different diseases like Dengue, Ebola, Influenza, and Malaria along with other infectious diseases (15). Further, a time series will be called stationary if it possesses the statistical properties that mean, variance, autocorrelation and other parameters are stationary over time. The stationarity of a time series is important for the statistical point of view as the regression coefficients are not Best Linear Unbiased Estimates (BLUE) for the non-stationary time series regression model because of the problem of autocorrelation, heteroscedasticity. A time series is known as strongly stationary or strictly stationary if it has fixed mean, fixed variance, and fixed covariance over fixed time intervals while a time series is known as weakly stationary, or second-order stationary if it has fixed mean and the covariance independent of time but depending on the size of the fixed time intervals, which is known as autocovariance function. Now the evaluation of the best fitted time series model for prediction precision and comparison of different fitted models, there are various measures like the coefficient of determination, MSE (Mean Squared Error), MAD (Mean Absolute Deviation), RMSE (Root Mean Squared Error), MAE (Mean Absolute Error) or MAPE (Mean Absolute Percentage Error), AIC (Akaike Information Criteria), BIC (Bayesian Information Criteria), Theil's U-statistics, etc.

Now we will discuss the different times series regression models used in epidemiology one by one in detail but as stationary is one of the important parts of the time series regression modeling, so we will first understand what is stationarity and non-stationarity in mathematical forms.

### Stationary Time Series

A time series {*Y*_*t*_, *t* ∈ *N*(Set of Natural Numbers)} is called strongly stationary or strictly stationary if it is independent of time difference that is,

$$\begin{array}{l} \left(Y_{t_{1}},Y_{t_{2}},\ldots ,Y_{t_{n}}\right)≅\left(Y_{t_{1}+h},Y_{t_{2}+h},\ldots ,Y_{t_{n}+h}\right) \end{array}$$

Where (*t*_1_, *t*_2_, …, *t*_*n*_) are different time points and *h* is a positive integer.

That is the joint distribution of (*Y*_*t*_1__, *Y*_*t*_2__, …, *Y*_*t*_*n*__) is identical to that of (*Y*_*t*_1_+*h*_, *Y*_*t*_2_+*h*_, …, *Y*_*t*_*n*_+*h*_) for all *h*. Thus, the joint distribution of (*Y*_*t*_1__, *Y*_*t*_2__, …, *Y*_*t*_*n*__) is invariant over time.

A time series {*Y*_*t*_, *t* ∈ *N*} is called weakly stationary or second-order stationery if it has fixed mean *E*(*Y*_*t*_) = μ and covariance (*Y*_*t*_, *Y*_*t*+*h*_) = γ_*h*_, where μ is fixed and γ_*h*_ is independent of time *t*. The sequence {γ_*h*_, *h* ∈ *N*} is known as the autocovariance function. The autocorrelation function (ACF) is also defined as ρ_*h*_ = γ_*h*_/γ_0_ = Corr(*Y*_*t*_, *Y*_*t*+*h*_). Naturally, a strictly stationary time series is weakly stationary. If the time series follows the normal distribution, then (*Y*_*t*_1__, *Y*_*t*_2__, …, *Y*_*t*_*n*__) is multivariate normal for all time points (*t*_1_, *t*_2_, …, *t*_*n*_) and then weak stationarity tends to strong stationarity. γ_0_ = Var(*Y*_*t*_) > 0 with the assumption that *Y*_*t*_ is a random variable and γ_*h*_ is symmetric in nature that is γ_*h*_ = γ_−*h*_ for all *h*.

### AR Model

As we are dealing with the single variable that is number of infections and we are interested in predicting this number. Thus, the time series regression models where the previous values of a variable are used as the regressors, are the autoregressive models. The autoregressive time series regression model of order *p*, developed by Yule (16) is signified by AR(*p*) is given by,

$$\begin{array}{l} Y_{t}=\theta_{1}Y_{t-1}+\theta_{2}Y_{t-2}+\ldots +\theta_{p}Y_{t-p}+\varepsilon_{t} \end{array}$$

or,

(1) $$\begin{array}{r} Y_{t}=\overset{p}{\underset{r=1}{\sum }}\theta_{r}Y_{t-r}+\varepsilon_{t} \end{array}$$

where θ_1_, θ_2_, …, θ_*p*_ are the fixed constants and ε_*t*_ is the error involved in the model associated with the observation *Y*_*t*_ for the time point *t* and which are independently distributed with 0 mean and fixed variance σ^2^.

The first order autoregressive model AR (1) is defined by,

(2) $$\begin{array}{r} Y_{t}=\theta_{1}Y_{t-1}+\varepsilon_{t}=\varepsilon_{t}+\theta_{1}Y_{t-1} \end{array}$$

To find the autocovariance function of the above model, we put the successive values and get,

$$\begin{array}{l} Y_{t}=\varepsilon_{t}+\theta_{1}\left\{\varepsilon_{t-1}+\theta_{1}\left(\varepsilon_{t-1}+\ldots \right)\right\}=\varepsilon_{t}+\theta_{1}\varepsilon_{t-1}+\theta_{1}^{2}\varepsilon_{t-2}+\ldots \end{array}$$

The time series {*Y*_*t*_} is called stationary of order two if *E*(*Y*_*t*_) = 0 and its auto covariance function is given by,

$$\begin{array}{l} \gamma_{0}=E\left(Y_{t}^{2}\right)-{\left\{E\left(Y_{t}\right)\right\}}^{2}=E\left(Y_{t}^{2}\right)\\ =E{\left(\varepsilon_{t}+\theta_{1}\varepsilon_{t-1}+\theta_{1}^{2}\varepsilon_{t-2}+\ldots \right)}^{2}\\ =\left(1+\theta_{1}^{2}+\theta_{1}^{4}+\ldots \right)\sigma^{2}=\frac{\sigma^{2}}{1-\theta_{1}^{2}} \end{array}$$

and

$$\begin{array}{l} \gamma_{h}=E\left(\overset{\infty }{\underset{r=0}{\sum }}\theta_{1}^{r}\varepsilon_{t-r}\overset{\infty }{\underset{s=0}{\sum }}\theta_{1}^{s}\varepsilon_{t+h-s}\right)=\frac{\sigma^{2}\theta_{1}^{h}}{1-\theta_{1}^{2}} \end{array}$$

Alternatively, these results may be obtained more simply as,

Multiplying both sides of equation (2) by *Y*_*t*−*h*_ and taking expectation on both sides, we get,

$$\begin{array}{l} E\left(Y_{t}Y_{t-h}\right)=E\left(\theta_{1}Y_{t-1}Y_{t-h}\right)+E\left(\varepsilon_{t}Y_{t-h}\right) \end{array}$$

Thus, the recurrence relation for the autocovariance may be written as,

$$\begin{array}{l} \gamma_{0}=\theta_{1}\gamma_{h-1},h=1,2,\ldots \end{array}$$

Squaring equation (2) on both sides and taking expectations on both sides, we have

$$\begin{array}{l} E\left(Y_{t}^{2}\right)=\theta_{1}^{2}E\left(Y_{t-1}^{2}\right)+2\theta_{1}E\left(Y_{t-h}\varepsilon_{t}\right)+E\left(\varepsilon_{t}^{2}\right)\\ \;=\theta_{1}^{2}E\left(Y_{t-1}^{2}\right)+\sigma^{2},\;as\;E\left(Y_{t-h}\varepsilon_{t}\right)=0 \end{array}$$

And thus we have,

$$\begin{array}{l} \gamma_{0}=\frac{\sigma^{2}}{\left(1-\theta_{1}^{2}\right)} \end{array}$$

Now the autocorrelation function may also be obtained for the model in (1) by multiplying the equation (1) on both sides by *Y*_*t*−*h*_ and taking expectations on both sides, we get the Yule-Walker equations after dividing the whole equation by γ_0_. Thus, we have,

(3) $$\begin{array}{r} \rho_{0}=\theta_{1}\rho_{h-1}+\theta_{2}\rho_{h-2}+\ldots .+\theta_{p}\rho_{h-p},\;h=1,2,\ldots \end{array}$$

Equation (3) is the recurrence relation for the autocorrelation function and the general solution for the relation (3) is,

(4) $$\begin{array}{r} \rho_{h}=C_{1}w_{1}^{\left|h\right|}+C_{2}w_{2}^{\left|h\right|}+\ldots +C_{p}w_{p}^{\left|h\right|} \end{array}$$

where *w*_1_, *w*_2_, …, *w*_*p*_ are the roots of the equation,

(5) $$\begin{array}{r} w^{p}-\theta_{1}w^{p-1}-\theta_{2}w^{p-2}-\ldots \;\theta_{p}=0 \end{array}$$

with the *C*_1_, *C*_2_, …, *C*_*p*_ is obtained from the relations ρ_0_ = 1 and from the equations for *h* = 1, 2, …, *p* − 1.

It is obvious that for *h* → ∞, γ_*h*_ → 0 which is possible if the roots lie inside the unit circle |*w*_*i*_| < 1, therefore the values of θ_1_, θ_2_, …, θ_*p*_ to be chosen are restricted.

### MA Model

The residual error terms in a time series is another source of information which is of paramount importance for model prediction. The residual errors themselves form a time series that can have temporal structure which in turn can be used to correct forecasts. The model is known as a moving average model developed by Eugen (17), bearing same name but different from moving average smoothing method. Auto regressive model is used when *Y*_*t*_ depends on its some lagged values while there are cases when *Y*_*t*_ depends on the random error term ε_*t*_ and its lagged values, which is supposed to be white noise that is, ε_*t*_ is independently and identically distributed (i.i.d) with 0 mean and fixed variance σ^2^. The MA model differs from AR model in two ways, one MA model propagate to forthcoming values of the time series outright for instance ε_*t*−1_ directly is on right hand side of *Y*_*t*_ equation. Thus, MA (*q*) model is the model with *q* lagged values of the error terms known as the MA model of order *q* is given by,

(6) $$\begin{array}{r} Y_{t}=\phi_{0}\varepsilon_{t}+\phi_{1}\varepsilon_{t-1}+\phi_{2}\varepsilon_{t-2}+\ldots +\phi_{q}\varepsilon_{t-q} \end{array}$$

where, ϕ_0_ = 1 and ϕ_1_, ϕ_2_, …, ϕ_*q*_ are the constants and error terms are supposed to be white noise. The estimates of the parameters or constants are obtained by the well-known method of least squares and it may be observed that the above MA model is stationary at second-order that is,

$$\begin{array}{l} \gamma_{h}=\{\begin{array}{ll} 0, & |h|>q\\ \sigma^{2}\overset{q-|h|}{\underset{r=0}{\sum }}\phi_{r}\phi_{r+h} & |h|<q \end{array} \end{array}$$

Further, it is to be worth notable that two MA models may have the same autocorrelation function. For example, let us consider two MA (1) models as,

$$\begin{array}{l} Y_{t}=\varepsilon_{t}+\phi \varepsilon_{t-1}\;and\;Y_{t}=\varepsilon_{t}+\phi^{-1}\varepsilon_{t-1},\;where,\;\phi^{-1}=1/\phi \end{array}$$

For both the models for |*h*| > 1, we observe that $\rho_{1}=\frac{\phi }{1+\phi^{2}}$ and ρ_*h*_ = 0, however from the model

$$\begin{array}{l} Y_{t}=\varepsilon_{t}+\phi \varepsilon_{t-1}, \end{array}$$

we have,

$$\begin{array}{l} \varepsilon_{t}=Y_{t}-\varepsilon_{t}+\phi \varepsilon_{t-1}\\ \;=Y_{t}-\phi \left(Y_{t-1}-\phi \varepsilon_{t-2}\right)=Y_{t}-\phi Y_{t-1}-\phi^{2}Y_{t-2}-\ldots \end{array}$$

Which is representing a tailor series expansion and which is only valid with the condition that |ϕ| < 1. Thus, we may observe that the error term ε_*t*_ at a time point *t* is represented in terms of the lagged values of the variable *Y* under study and hence we may say it is an invertible model and it is to be mentioned that no two invertible models have the same autocorrelation function.

### ARMA Model

The autoregressive moving average ARMA of order (*p, q*) is obtained by combining the AR (*p*) model of order *p* and the MA(*q*) model of order *q*. Autoregressive Moving Average (ARMA) models, describe a weak stationary random process in a couple of polynomials, one for the autoregression (AR) and another for the moving average (MA). For a time series *Y*_*t*_, the ARMA model predicts the future values of the series. The AR part regresses the variable for its own lagged values. The MA part takes into account the error terms which occur synchronously at different time points in the past. ARMA is suitable for unobserved shocks as in case of pandemic and it is due to the MA or moving average along with its behavior. The ARMA (*p, q*) model introduced by Wold (18) is presented as,

$$\begin{array}{l} Y_{t}=\left(\theta_{1}Y_{t-1}+\theta_{2}Y_{t-2}+\ldots +\theta_{p}Y_{t-p}\right)\\ \;+\left(\phi_{0}\varepsilon_{t}+\phi_{1}\varepsilon_{t-1}+\ldots +\phi_{q}\varepsilon_{t-q}\right), \end{array}$$

or

(7) $$\begin{array}{r} Y_{t}=\overset{p}{\underset{r=1}{\sum }}\theta_{r}Y_{t-r}+\overset{q}{\underset{s=1}{\sum }}\phi_{s}\varepsilon_{t-s} \end{array}$$

Where, θ_1_, θ_2_, …, θ_*p*_ and ϕ_1_, ϕ_2_, …, ϕ_*q*_ along with ϕ_0_ = 1 and the error terms ε_*t*_ is white noise. Now the well-known Box and Jenkins (19) procedure may be applied for better forecasting as it uses an estimation of the parameters or constants of the model and provides the diagnostics of these parameters to the best. The estimation of the parameters of the ARMA model is done using the ordinary least square method.

To understand it better let us consider an example. Let {*X*_*t*_} be the unobserved sequence of observations and {*Y*_*t*_} be the observed sequence and we have,

$$\begin{array}{l} X_{t}=\theta X_{t-1}+\varepsilon_{t}\;and\;Y_{t}=X_{t}+\eta_{t} \end{array}$$

where, ε_*t*_ and η_*t*_ are independent and white noise and it is to be mentioned that {*X*_*t*_} is AR (1). Now we may write that,

$$\begin{array}{l} \xi_{t}=Y_{t}-\theta Y_{t-1}\\ \;=\left(X_{t}+\eta_{t}\right)-\theta \left(X_{t-1}+\eta_{t-1}\right)\\ \;=\left(X_{t}-\theta X_{t-1}\right)+\left(\eta_{t}-\theta \eta_{t-1}\right)\\ \;=\varepsilon_{t}+\eta_{t}-\theta \eta_{t-1} \end{array}$$

Now it may be observed that ξ_*t*_ is stationary and *Cov*(ξ_*t*_, ξ_*t*+*h*_) = 0, *h* ≥ 2. Thus, ξ_*t*_ may be modeled as MA (1) model and {*Y*_*t*_} as ARMA (1, 1) model. To find how much order should be taken for better forecasting that what should be the optimum values of *p* and *q*, respectively, we use the ACF (Autocorrelation Function) and PACF (Partial Autocorrelation Function) plot.

### ARIMA Model

The autoregressive integrated moving average (ARIMA) model, suggested Box et al. (20) is a generalization of the ARMA model with non-stationary series. ARIMA is non-stationary means that it has non-constant mean and variance over time. The integrated part refers to a differencing initial step, which can be applied to eliminate the non-stationarity of the series. Some application of this method to epidemiological time series may be in Promprou et al. (21), Liu et al. (22), and Coutin (23).

An ARIMA model is unequivocal by its three components:

- *Auto regression (AR)* model is the model which represents a variable that regresses on its lagged, or prior, values.
- *Integrated (I)* shows the differencing of basic observations so that the time series may be stationary.
- *Moving average (MA)* provides the docility between an observation and a residual from the MA model for lag observations.

Let {*Y*_*t*_} be the time series that is not stationary. Now we make it stationary through the differencing method and see the order of differencing which makes the series stationary. Let the first-order difference of the series is,

$$\begin{array}{l} X_{t}=\nabla Y_{t}=Y_{t}-Y_{t-1} \end{array}$$

The second-order differences are,

$X_{t}=\nabla^{2}Y_{t}=\nabla \left(\nabla Y_{t}\right)=Y_{t}-2Y_{t-1}+Y_{t-2}$ and so on. Whenever we observe that the differencing process is stationary, we say that the time series is stationary and we may go for the ARMA model for this integrated series. Thus, {*Y*_*t*_} is known as an ARIMA (autoregressive integrated moving average) model, denoted by ARIMA(*p, d, q*) if $X_{t}=\nabla^{d}Y_{t}$ is a ARMA(*p, q*) model.

### SARIMA Model

**SARIMA** is nothing but seasonal **ARIMA** and is suitable for the time series with seasonality. Seasonal Autoregressive Integrated Moving Average (SARIMA) or Seasonal ARIMA, is an extended version of ARIMA representing a univariate series with the new seasonal component. There are three new hyperparameters as AR, differencing (I) and MA for the seasonal component of the series with an additional seasonality parameter. The SARIMA model is denoted by ARIMA(P, *D, Q*)_*m*_, where *m* represent observations per year and the block letters are for the seasonal parts of the model, and small letters for the non-seasonal parts. The parameters of the ARIMA models can be estimated through the Box-Jenkins approach.

An overview of the Box-Jenkins Methodology involves the three steps given below:

1. Identification. Through scatter plot, autocorrelations, partial autocorrelations, and other knowledge. Then a family of ingenious ARIMA models has opted and estimation of p, d, and q is done.
2. Estimation. The model parameters phis and thetas under consideration are estimated through well-known maximum likelihood method (MLE), backcasting, and others as discussed by Box and Jenkins (19).
3. Diagnostic Checking. The inadequacy of the fitted model is checked through autocorrelations of the residuals or error, values.

These steps are kept on iteratively till the diagnostic procedure does not provide improvement in the model. The basic methodology is to get differences between data points to get an outcome that is to make it stationary. The methodology permits the model to recognize the trends through AR, MA, and seasonal differencing for forecasting. ARIMA models are one of the types of Box-Jenkins model. The terms ARIMA and Box-Jenkins Model may be used equivalently.

ARIMA is a statistical technique based on observations rather than theory, while compartmental models are essential mechanistic mathematical models based on biological laws. In the further sections of the current review, we have also discussed such biological law based models in detail. Apart from these, many more advanced and efficient computational techniques with mechanistic mathematical models are now in use for the forecasting of the spread of the various infectious diseases including COVID-19. These advanced computational techniques includes ETS (error, trend, seasonal) state space models which comes under the category of exponential smoothing method, STLM (seasonal and trend decomposition using losses) method, TBATS (trigonometric, box-cox, ARMA, trend, seasonal) method, FASSTER (forecasting with additive switching of seasonality, trend and exogenous regressors) model which is used to capture patterns of multiple seasonality in a state space framework by using state switching, neural network method, deep learning method using artificial neural networks (ANN), long short-term memory (LSTM) neural network method and hybrid model such as support vector regression (SVR) method for the explanation and forecasting of the outbreak of infectious diseases including COVID-19 disease. Almost all these advanced computational techniques make use of regression analysis with some additional features.

The above time series regression models along with the advanced computational techniques have been used by various researchers for a better understanding and the forecasting of different infectious diseases. Some of the latest contributions are presented in Table 3.

**Table 3:** Different time series regression models for different infectious diseases.

**S. no**.	**References**	**Conclusion drawn**
1	Zhang et al. (15)	In this study, the authors have presented a complete analysis of different predicting methods based on the monthly infection spread data of typhoid fever. The seasonal autoregressive integrated moving average (SARIMA) model and three different models inspired by neural networks, namely, backpropagation neural networks (BPNN), radial basis function neural networks (RBFNN), and Elman recurrent neural networks (ERNN) were compared. The dissimilarities, pros, and cons, between the two models. The evaluation was based on three metrics: mean absolute error (MAE), mean absolute percentage error (MAPE) and mean square error (MSE). The results showed that RBFNN obtained the smallest MAE, MAPE, and MSE in both the modeling and forecasting processes. Ultimately, it was suggested to use the RBFNN method for better explanation and prediction of typhoid fever infection spread.
2	Zhang et al. (74)	In this work, nine types of infections were compared based on the efficiency of four-time series methods, regression and exponential smoothing, ARIMA, and support vector machine (SVM). The performances were evaluated based on three metrics: mean absolute error (MAE), mean absolute percentage error (MAPE) and mean square error (MSE). The robustness of the statistical models in predicting the potential spread of the infections showed their good application in epidemiological surveillance and found that no single method is completely superior to the others but support vector machine-based methods are proven better than the ARIMA models and decomposition methods in most of the cases.
3	Imai et al. (75)	In this study, time series regression was applied to evaluate the short-term associations of air pollution and weather with mortality or morbidity of infectious diseases. They used different approaches, including mathematical modeling, wavelet analysis, and ARIMA models. They concluded that the time series regression can be used to investigate the dependence of infectious diseases on weather, but may need modifying to allow for features specific to this context.
4	Song et al. (76)	The authors compiled monthly data of influenza infections from all provinces and autonomous regions in mainland China and applied the time series analysis to construct an ARIMA model. They have evaluated the goodness of fit through Autocorrelation function (ACF), partial autocorrelation function (PACF), and automatic model selection was to determine the order of the model parameters. It is conceivable that SARIMA is the best time series model for the prediction of influenza infection spread.
5	Sarkar and Chatterjee (77)	The authors have applied different time series models to analyze and forecast financial data as well as epidemiological data of malaria infection dissemination. They have studied epidemiological data of malaria using three-time series models, namely Auto-Regressive Integrated Moving Average (ARIMA), Generalized Auto-Regressive Conditional Heteroskedastic (GARCH), and Random Walk. They have shown a good fit of models on the data and provided the best forecast for future infection spread. As far as future prevalence pattern is concerned, the prediction of these models may help researchers and public health professionals to design control programs for malaria.
6	Chae et al. (78)	The authors studied the prediction of infections by optimizing the parameters of deep learning algorithms while considering big data including social media data. The performance of the deep neural network (DNN) and long-short term memory (LSTM) learning models were compared with the autoregressive integrated moving average (ARIMA) when predicting three infections for 1 week time into the future. They have shown that the DNN and LSTM models perform better than ARIMA. The DNN model performed stably and the LSTM model was more accurate when infections were spreading.
7	Tapak et al. (79)	The author analyzed the correctness of support vector machine, artificial neural network, and random-forest time series models in influenza-like illness (ILI) modeling and infection detection. Different models were applied to a data set of weekly ILI cases data in Iran. To judge the robustness of the models, the root means square errors (RMSE), mean absolute errors (MAE), and intra-class correlation coefficient (ICC) calculations were used as testing criteria. It was indicated that the random-forest time series model worked better in comparison to the rest three methods. The outcome depicted that the used time series models had excellent performance suggesting these could be effectively applied for predicting weekly ILI infections and endemics.
8	Chaurasia and Pal (80)	In this work, the authors have analyzed the number of cases, deaths, and recovery cases in the case of COVID-19 worldwide within a specific period. They have used several prediction techniques: naive method, simple average, moving average, single exponential smoothing, Holt linear trend method, Holt-Winters method, and ARIMA, for comparison, and how these methods improve the Root mean square error score. They concluded that the naive method is best in comparison to other used methods.
9	Rahmadani and Lee (81)	The authors suggested a hybrid deep learning framework using the meta-population model and long and short term model (LSTM) for the prediction of the COVID-19 dissemination. They expanded the susceptible–exposed–infected–recovered compartment model by taking into account the human mobility among a number of regions. They used the meta-population model to incorporate with deep learning models to estimate the parameters of the combined hybrid model. They have compared the suggested hybrid deep learning framework with other estimation methods for the prediction of COVID-19 spread patterns and have shown improvement over previously presented methods.
10	Kalantari (82)	The author used the singular spectrum analysis (SSA) method for the prediction of the number of daily confirmed infection cases, deaths, and recoveries caused by COVID-19. It was analyzed using SSA method with the other commonly used time series predicting techniques including ARIMA, fractional ARIMA, exponential smoothing, TBATS, and neural network autoregression (NNAR) on the basis of fitting measure root mean squared error (RMSE). It was shown that the SSA technique is best for predicting the number of daily confirmed infection cases, deaths, and recoveries caused by COVID-19 among the studied models.
11	Satrio et al. (83)	The authors utilized the machine learning model for predicting the spread of COVID-19 in Indonesia. They have also attempted to estimate a time line for the return of the normalcy. They have utilized PROPHET forecasting model as well as ARIMA to see their robustness and accuracy for the confirmed new infection cases, deaths, and recovered numbers. They have shown that PROPHET performs better than ARIMA model on the analyzed data set.
12	Beneditto et al. (83)	The authors utilized the Machine Learning model to forecast the trend of the disease in Indonesia with finding out the approximation when normality will return. This study used Facebook's Prophet Forecasting Model and ARIMA Forecasting Model to compare their performance and accuracy on a dataset containing the confirmed cases, deaths, and recovered numbers, obtained from the Kaggle website. The prediction models are then compared to the last 2 weeks of the actual data to measure their performance against each other. The result showed that Prophet has predicted the outcomes better than ARIMA, despite it being further from the actual data the more days it predicts.

## Epidemiological Modeling

Epidemiology is the science of the study of the occurrence of the disease. It is an unusually large and/or short-term spread of the disease and is known as endemic if it outstays in a population. As it is an infectious disease, so its spread is not only because of disease factors like the infectious agent, mode of transmission, latent period, infectious period, susceptibility, and resistance but also due to social, culture, demographic, economic, and geographic factors (24). Epidemiology also determines different groups of individuals in the population based on similar characteristics like sex, age, size, etc. while ignoring the uniqueness of an individual. It determines whether the divisions of the individuals in the population into different groups tell something more than what we could get from each individual separately. Through epidemiological modeling, the aim is to describe, analyze and understand the patterns of infectious disease in these groups.

Before discussing the epidemiological models, we will first discuss the classification of the diseases based on their agents and medium of transmission. Table 4 represents the classification of the diseases adapted and modified from Hethcote (24) based on their agents and medium of transmission.

**Table 4:** Different infectious diseases their causative microorganisms and modes of transmission.

**Microorganism**	**Mode of transmission**			
	**Person → person**	**Person-environment** **environment → person**	**Reservoir → vector vector → person**	**Reservoir → person**
Virus	Measles, Chickenpox, Mumps, Rubella, Smallpox, Influenza, Herpes, HIV (AIDS virus)	Poliomyelitis	Arboviruses: Yellow fever Dengue fever Encephalitis Tick fever Sandfly fever	Rabies
Bacteria	Gonorrhea Tuberculosis Pneumonia Meningitis Strep Throat	Typhoid Fever Cholera	Plague	Brucellosis Tularemia Anthrax
Protozoa	Syphilis	Amebiasis	Malaria Trypanosomiasis	
Helminths			Schistosomiasis Filariasis Onchocerciasis	Trichinosis

Apart from chronic diseases like cancer, heart attack, and diabetes, infectious diseases are a very common reason for deaths in the whole world. The immunodeficiency virus (HIV) that may confer Acquired Immunodeficiency Syndrome (AIDS), Ebola, SARS, Dengue, Tuberculosis, and currently COVID-19 among others have become very crucial infectious diseases for the whole world. The transmission mechanism of the infectious diseases from the infective to susceptive through the chain of infections is of crucial importance. So it becomes necessary to model the diseases so that their transmission mechanism may be revealed and effective policies may be formed to control or overcome the infectious disease. As the transmission interaction of an infectious disease in the population is a very complicated process, so it is very tuff to understand its dynamics without mathematical or statistical modeling. An epidemiological model is used to forecast the macroscopic behavior of infectious disease outbreaks by a population through microscopic description that is the role of an individual infectious (25).

The epidemiological modeling is also done to know the competing risks of the deaths from infectious diseases. Modeling also attempts to limit the extent of the infection employing some suppressive strategies like quarantining, social distancing, culling in animals, contact tracing, and vaccination when it is available. One of the weaknesses of the modeling is that the data are limited for infectious diseases and it is many times unethical to experiment on humans, so we must go for the optimal combination and use the available resources. Epidemiological modeling is crucial to know the salient features of the infection dynamics of the disease. The forecast or prediction of the outcomes of the diseases in communities from the changes in demographics, community structure, disease characteristics, and suppressive strategies imposed on it.

In the eighteenth century, Bernoulli (26), who was the pioneer scientist in the field during the 18th century formulated and analyzed the epidemiological model for smallpox. Through his model, he evaluated the effectiveness of the vaccination inoculation of healthy people against the smallpox virus. He has obtained mathematical data on this issue to influence public health policy by encouraging the universal vaccination for smallpox. His work was first presented at the Royal Academy of Sciences in Paris in 1760 and later published in 1766. Hamer (27) proposed and analyzed a discrete-time epidemiological model and concluded the recurrence of measles. He has pointed out about the germinable source, it has been suggested that periodic evolutionary changes in the life history of microorganisms may explain the waves of disease, but is the periodic manifestation by the micro-organisms or the interaction between the microbe and the host tissues. Ross (28) suggested a model with differential equations for analyzing the mechanism of malaria as a host-vector disease. He has mentioned that it may not be fatal, although its wide prevalence in almost all warm climates results in the aggregate a large amount of sickness and deaths globally. Further, he has presented the real-time data representing only in India the official estimate of the means annual death rate of five per thousand that mean on average one million, one hundred thirty thousand deaths every year. He observed that the mortality by malaria is more than the plague, cholera, and dysentery altogether. Kermack and McKendrick (29) extended the model of Ross (28) and obtained the threshold results of the malaria epidemic. Kermack and McKcndrick (29) analyzed different epidemiological models and contributed to the mathematical theory of epidemics. They have observed that one of the most crucial characteristics in the study of the spreading infections is the difficulty of finding a causal factor that appears to be enough to account for the scale of the recurrent infection waves of disease which contract every population. They asked for extracting more details of the effects of the various factors which may affect the spread of the infections. Cassels et al. (30) analyzed the mathematical models for HIV transmission dynamics. Cohen et al. (31) analyzed the mathematical modeling of Tuberculosis transmission dynamics. Andraud et al. (32) studied the dynamic epidemiological models for dengue transmission and provided a systematic review of different structural approaches. Taghikhani and Gumel (33) worked on the mathematics of dengue transmission dynamics and explained the roles of vector vertical transmission and temperature fluctuations. Xia et al. (34) studied and discussed the modeling of transmission dynamics of Ebola virus disease in Liberia while Agusto (35) studied the mathematical model dynamics of transmission of Ebola with decline and re-infection. Some of the latest contributions are presented in Table 5.

**Table 5:** Various epidemiological models for different infectious diseases.

**S. no**.	**References**	**Conclusion drawn**
1	Huppert and Katriel (84)	The authors have discussed the extent to which the disease transmission models provide reliable predictions. They examined the predictions of the model to test which are trustworthy. An important benefit derived from mathematical modeling activity is that it demands transparency and accuracy regarding our assumptions, thus enabling us to test our understanding of the disease epidemiology by comparing model results and observed patterns. Models can also assist in decision-making by making projections regarding important issues such as intervention-induced changes in the spread of disease.
2	Steele et al. (85)	The authors mentioned that the early detection of infectious disease outbreaks can reduce the ultimate size of the outbreak, with lower overall morbidity and mortality due to the disease. In the review, they have mentioned numerous approaches to the earlier detection of outbreaks exist. In the systematic review the authors used of PRISMA framework (Preferred Reporting Items for Systematic Reviews and Meta-analyses), The MEDLINE (PubMed) database. Five studies were identified and included in the review. These studies evaluated the effect of electronic-based reporting on detection timeliness, the impact of laboratory agreements on timeliness, and barriers to notification by general practitioners.
3	Driessche (86)	The author worked on the basic reproduction number, **R**_0_, for infectious diseases, and other reproduction numbers related to **R**_0_ that are useful in guiding control strategies. Beginning with a simple population model, the concept is developed for a threshold value of **R**_0_ determining whether or not the disease dies out. The next generation matrix method of calculating **R**_0_ in a compartmental model is described and illustrated. These theoretical ideas are then applied to models that are formulated for West Nile virus in birds (a vector-borne disease), cholera in humans (a disease with two transmission pathways), anthrax in animals (a disease that can be spread by dead carcasses and spores), and Zika in humans (spread by mosquitoes and sexual contacts). Finally, references for other ways to calculate **R**_0_ are given and these are useful for more complicated models.
4	Walters et al. (87)	The authors observed that mathematical models can aid in the understanding of the risks associated with the global spread of infectious diseases. To assess the current state of mathematical models for the global spread of infectious diseases, the authors reviewed the literature highlighting common approaches and good practice, and identifying research gaps. They found that most epidemiological data come from published journal articles, population data come from a wide range of sources, and travel data mainly come from statistics or surveys, or commercial datasets. However, they believed that open access datasets should be used wherever possible to aid model reproducibility and transparency.
5	Raissi et al. (88)	The authors considered the compartmental disease transmission models and discuss the importance of determining model parameters that provide an insight into disease transmission and prevalence. They used three approaches including an optimization approach, a physics informed deep learning, and a statistical inference method to estimate parameters and analyze disease transmission. The performance of the deep learning method is validated against representative small and big data sets corresponding to a well-known benchmark example and the results indicate that deep learning is a viable candidate to determine model parameters. The results indicate the efficiency and importance of statistical inference methods for researchers to understand and analyze the data to make confident predictions.
6	Li et al. (36)	The authors established the dynamics model of infectious diseases and the time series model to predict the trend and short-term prediction of the transmission of COVID-19, in mainland China for clinical trials. They applied the dynamic models of the six chambers and established the time series models based on different mathematical formulas according to the variation law of the original data. Finally, they suggested that it is a very effective prevention and treatment method to continue to increase investment in various medical resources to ensure that suspected patients can be diagnosed and treated promptly.
7	Prasse et al. (89)	The authors have used a network-based model to describe the COVID-19 epidemic in the Hubei province. They have suggested the network-inference-based prediction algorithm (NIPA) to predict the future prevalence of the COVID-19 epidemic in the cities of China and they have shown that NIPA is best for accurate prediction of the infection spread.
8	Yang et al. (90)	The authors have described the short-term predictor of the daily cases reported in Wuhan City using individual-level network-based model to rebuilt the epidemic dynamics in Hubei Province and have seen the effectiveness of non-pharmaceutical interventions on the epidemic spreading with various scenarios. They have shown through the simulation study that without continued control measures, the epidemic in Hubei Province could have become persistent and the infection rate is controlled through protective measures and social distancing. They have demonstrated the COVID-19 transmission with non-Markovian processes and have shown how these models produce different epidemic trajectories, in comparison to Markov processes.
9	Popov and Nakov (91)	The authors worked on the epidemiological models of the spread of infectious diseases, including COVID-19. The models and simulations of an epidemic in the presence of quarantine and the moment of its termination have been made. They have pointed out that it is important to pinpoint the timing of the lifting of measures or their granting. They have shown through the proposed simulation model that the impact of group gatherings such as the beginning of the school year, holidays, and more, mass events on the epidemic picture. These studies are also relevant in the event of a mutation in the virus that will change the rate of spread.
10	Saraee and Silva (92)	In this review, the authors have compared studies that have used epidemiological models for disease forecasting and other models that have identified socio-demographic factors associated with COVID-19. They have evaluated several models, from basic equation-based mathematical models to more advanced machine-learning ones. They have identified high-impact models used by policymakers and discussing their limitations, They have suggested possible areas of applications for future research.
10	Moein et al. (93)	The authors have used different mathematical techniques, including the susceptible-infected-recovered (SIR) model for the description and prediction of the infection spread of COVID-19. They have simulated the infection spread data in Isfahan province of Iran along with three suppressive measures of the stringency level of physical distancing. They have shown that for the short term prediction, SIR model was only able to predict the actual spread and pattern of COVID-19 while not in long term. They have also concluded that other published works using SIR models for predicting COVID-19 has the same drawback. The assumptions for SIR models are not true for COVID-19 pandemic. Finally they have suggested that more sophisticated modeling strategies and detailed knowledge of the biomedical and epidemiological aspects of the disease are needed to predict the spread of this pandemic.
11	Alvarez et al. (94)	The authors come up with a simple epidemiological model which may be implemented in Excel spreadsheets and able to simulate the data of the COVID-19 pandemic significantly. They have shown that the model may closely follow the evolution of COVID-19 spread in big cities by simply adjusting parameters of demographic conditions and aggressiveness of the response to epidemics. Further they have also advised that the suggested epidemiological simulator may be used to judge the efficiency of the response of population to the pandemic. The simplicity and accuracy of the model will help to understand the extent of an epidemic event and the efficacy of any policy response from the state.

Currently COVID-19 pandemic is going on worldwide and epidemiologists are trying to model its transmission mechanism and trying to predict various aspects so that better policies may be made to control the disease. Li et al. (36) analyzed the modeling and epidemic forecasting of COVID-19 and depicted its essentialness to pestilence anticipation and monitoring measures. Jewells et al. (37) discussed the modeling of the COVID-19 pandemic and described its theory and projection values for the US and worldwide. Ndaïrou et al. (38) worked on the mathematical modeling of COVID-19 transmission dynamics for the Wuhan city of China. Griffiths (39) pointed out and discussed whether mathematical modeling can solve the current COVID-19 crisis. Kucharski et al. (40) worked on the primary transmission dynamics and control of COVID-19 through mathematical modeling. Many authors worked and many are still working on the modeling of transmission dynamics of COVID-19 disease to predict it so that some concrete policies may be formed to control the crisis.

There are different epidemiological models applied to formulate the transmission dynamics of infectious diseases. Among them, SI (Susceptible and Infected) model, SIS (Susceptible, Infected and Susceptible) model, SIR (Susceptible, Infected and Recovered) model, SIRS (Susceptible, Infected, Recovered and Susceptible) model, SEIR (Susceptible, Exposed, Infected and Recovered) model, and SEIRS (SIR model with untested/unreported cases) model are some of the famous and frequently used epidemiological models for infectious diseases. Before going into the mathematics of these models, we will discuss the Basic Reproduction Number denoted by *R*_0_ which is very crucial for all epidemiological models along with the Effective Reproductive Number denoted by *R* and the Herd Immunity.

### The Basic Reproduction Number

*R*_0_ is a potential measure of the transmission of infectious disease and represents the mean number of secondary infections generated by a typical infected individual in a population with all susceptibles (41). *R*_0_ does not include the new cases generated by the secondary individuals. *R*_0_ is influenced by various factors as:

- The contact rate in the host population.
- The chance of infection during contact.
- The period of infectiousness.

It is worth notable that *R*_0_ is a pure number that is unit or dimension-free and may be represented as,

$$\begin{array}{l} R_{0}\propto \left[\frac{\text{Infection}}{\text{Contact}}\right]\times \left[\frac{\text{Contact}}{\text{Time}}\right]\times \left[\frac{\text{Time}}{\text{Infection}}\right]=\tau .\bar{c}.d \end{array}$$

Where, τ is the transmissibility or chance of infection during contact between susceptible and infected individuals. $\bar{c}$ being the mean contact rate between susceptible and infected individuals and *d* is the period of infectiousness.

Generally, an epidemic spreads in a susceptible population when *R*_0_ is more than one i.e., *R*_0_ > 1 and thus infection cases will increase. Rarely the entire population will be susceptible to be infected in the physical world. Some intromission will be immune may be because of prior infection which has developed life-long immunity, or maybe due to prior immunization. Therefore, the whole population will not be infected and the mean of secondary cases per infectious individual will be less than *R*_0_, and it is measured through the effective reproductive rate denoted by *R*. *R*_0_ depends on the disease and host population and it is different for different infectious diseases for instance *R*_0_ = 2.6 for TB in cattle, *R*_0_ = [3 4] for influenza in humans. *R*_0_ = [3.5 6] for smallpox in humans and *R*_0_ = [16 18] measles in humans. For more details on *R*_0_, please refer to Khajanchi et al. (42).

### The Effective Reproductive Number

R is the mean number of secondary infections per infectious case in a population under consideration for susceptible and non-susceptible hosts. The number of infectious cases increases with *R* > 1, like the beginning of an epidemic, while the infectious cases diseases for *R* = 1, and with *R* < 1, the number of infected cases will decline. *R* can be estimated by the product of *R*_0_ and the fraction susceptible (*X*) host population. Thus, *R* is defined as,

$$\begin{array}{l} R=R_{0}.X \end{array}$$

For instance, if *R*_0_ for influenza is 12 for any population where 50% of the population is immune, *R* for influenza will be 12 x 0.5 = 6. In such situations, a single influenza case would generate a mean of 6 new secondary infections. Thus, for the successful elimination of an infectious disease from the entire population, *R* must be <1 i.e., *R* < 1.

### Herd Immunity

Herd immunity develops in the population if a reasonable part of that population or herd gets vaccinated or immune through some mechanism, which results the protection of susceptible persons who are not vaccinated. The larger the portion of immune persons in the population, lower the chance to be infected. The diseases hardly spread from one individual to another if huge numbers are already immune since the transmission infection chain is broken. The herd immunity threshold is crucial for the population that should be immune so that the disease may be stable in the particular group. If it is attained, say by immunization, then every individual produces only one new case i.e., *R* = 1 and then the infection will be stable for the population. The Herd Immunity Threshold for a population is defined as,

$$\begin{array}{l} \text{HIT}=\frac{R_{0}-1}{R_{0}}=1-\frac{1}{R_{0}} \end{array}$$

Thus, if the threshold for herd immunity is outdoing, then *R* < 1 and infection cases decrease. Hence it is a crucial measure for infectious disease control, immunization, and eradication programs.

Now we will discuss different epidemiological models along with the mathematical dynamics and the transmission of infectious diseases.

### SI and SIS Models

**Figure 6:**
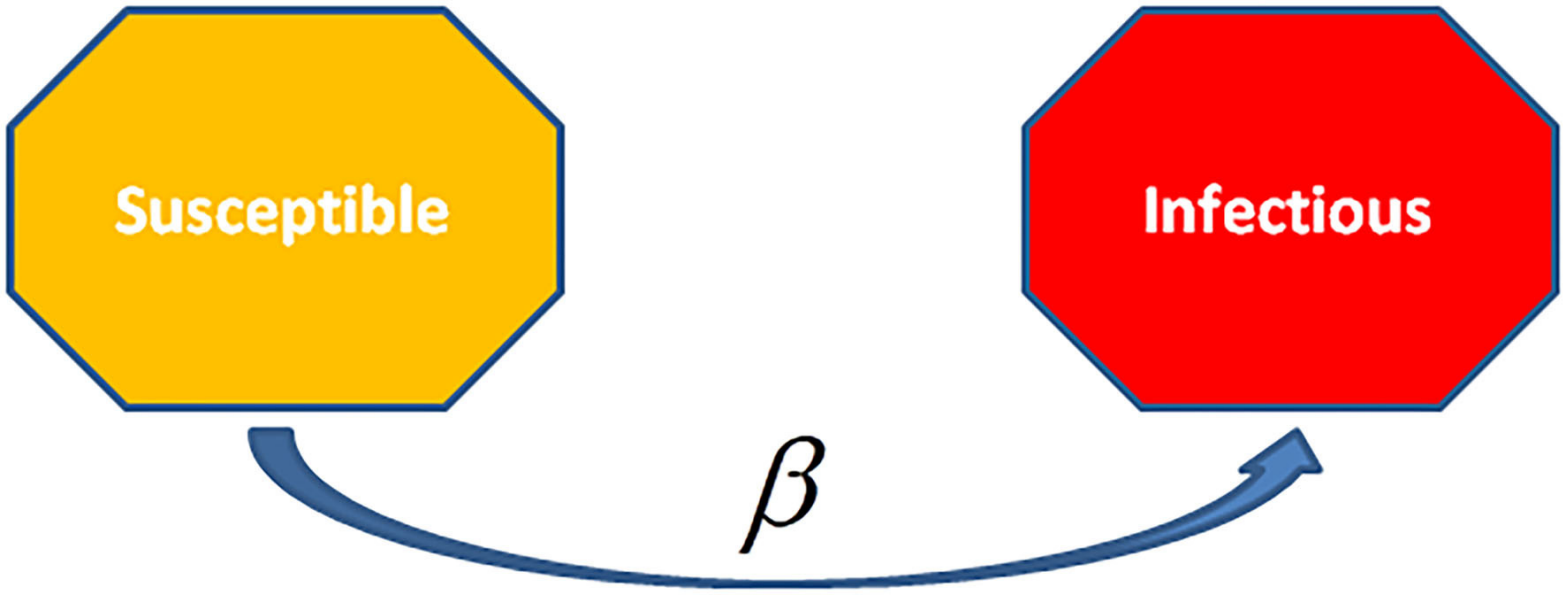
The Susceptible- Infectious (SI) Model is the ingenious model among the disease models. Units are born without immunity which means they are susceptible to all infections. When they will be infected and not given any treatments, then all cases remain life-long infected, and they remained in contact with the susceptible population. This model applies to diseases such as cytomegalovirus (CMV) and herpes.

The SI model is described by the differential equations which govern the deterministic SI compartmental model. In this model, people are always in the infectious state with lifelong infections, such as herpes disease has lifelong infectiousness (Figure 6). Han (43) worked on this model based on two-dimension small-world networks with epidemic alert. This technique of modeling is impractical to animal or human infections as in such case it is supposed that an infected unit will remain in the same state. The following diagram of the SI model represents how cases transmit from one compartment to another in the model. The curved line depicts the way the model transforms to an SIS (Susceptible-Infectious-Susceptible) model, where infection does not lead to immunity or say waning immunity. Persons have reinfections, and infected persons move to the susceptible state. For instance, sexually transmitted diseases (STD), like gonorrhea or chlamydia.

The infectious rate, β, checks over the spread rate, representing the chance of transmission of disease from an infectious individual to a susceptible one. Recovery rate, $\gamma =1/\bar{d}$, is the mean time, $\bar{d}$, of the infection. The SI model is ingenious among various infectious disease models. Cases are born in simulation having no immunity or susceptibility. Once the individual is infected and is without treatment, it remains infected long-life and remains with the susceptible population. This model is used for infectious diseases like cytomegalovirus (CMV) or herpes.

### SI Without Vital Dynamics

The transmission dynamics of I in a SI model behave like logistic growth. If no birth and death occur, then every susceptible individual will be infected. Be that as it may, it very well may be adjusted to a SI model by killing brooding and setting the irresistible duration to be prolonged as compared to human life expectancy. The SI model may be represented by Ordinary Differential Equations (ODE) as,

$$\begin{array}{l} \frac{dS}{dt}=-\frac{\beta SI}{N}\\ \frac{dI}{dt}=\frac{\beta SI}{N}=\beta I\left(1-\frac{I}{N}\right) \end{array}$$

Where, *N* = *S* + *I* is the whole population or *N*(*t*) = *S*(*t*) + *I*(*t*) is the whole population at the time *t*.

### SI With Vital Dynamics

Let μ and υ are the birth and death rates, for the SI model, respectively, with vital dynamics. For a population to be constant, we assume that μ = υ. Thus, the Ordinary Differential Equations for the SI model for this case are,

$$\begin{array}{l} \frac{dS}{dt}=\mu N-\frac{\beta SI}{N}-\upsilon S\\ \frac{dI}{dt}=\frac{\beta SI}{N}-\upsilon I \end{array}$$

Where, *N* = *S* + *I* is the total population or *N*(*t*) = *S*(*t*) + *I*(*t*) is the whole population at the time *t*.

The ultimate part of infected individuals is concerned with the vital dynamics μ, υ and β. The rate of infection β can be calculated from the steady-state as,

$$\begin{array}{l} \mu =\upsilon =\frac{\beta S}{N}=\beta \left(1-\frac{I}{N}\right) \end{array}$$

### SIS Model

**Figure 7:**
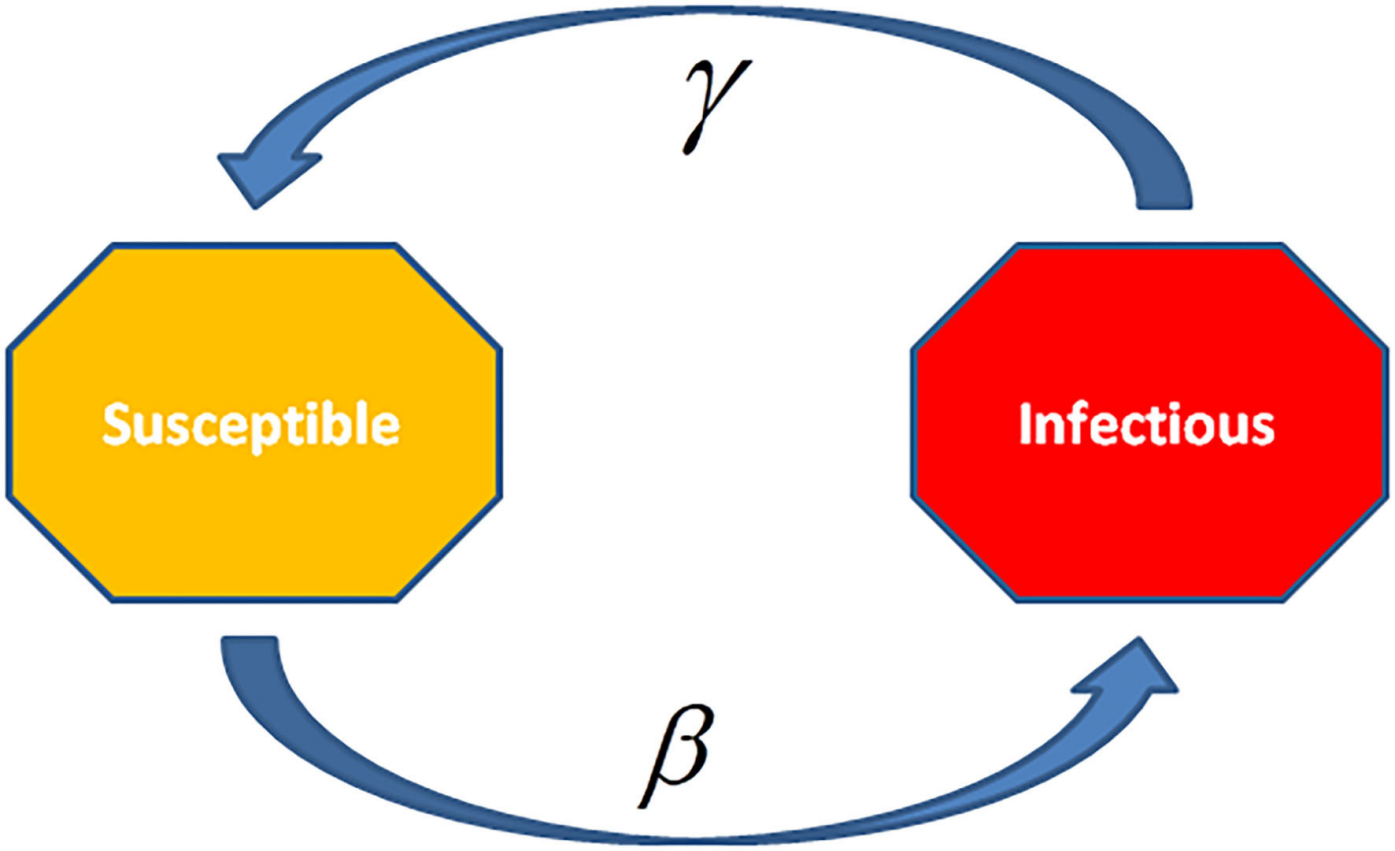
In Susceptible-Infectious-Susceptible (SIS) Model, the infected cases are again susceptible after recovery. This model is applied to the diseases, which have the common occurrence of re-infection and relapse cases, e.g., common cold (rhinoviruses) or sexually transmitted diseases (STDs) such as Gonorrhea or Syphilis.

In the SIS model, the infected cases are again in a susceptible state since infection (Figure 7). Such type of models is applied for the diseases which generally have encored infections, for instance, the common cold (rhinoviruses) or STD like gonorrhea or chlamydia. In this model, the cases move randomly in a cycle of Susceptible-Infected-Susceptible. Therefore, only two states exist one and another susceptible as the individuals are again susceptible after recovery. Deletions due to death or acquired immunization are not considered in this model.

### SIS Without Vital Dynamics

Since persons in the SIS model stay susceptible since infection, the disease achieves stability in a population, even with no deaths and births. The Ordinary Differential Equations (ODE) for this model with no births and deaths may get the solution to know the dynamics of the disease. These equations may be written as,

$$\begin{array}{l} \frac{dS}{dt}=-\frac{\beta SI}{N}+\gamma I\\ \frac{dI}{dt}=\frac{\beta SI}{N}-\gamma I \end{array}$$

Where, *N* = *S* + *I* is the whole population or *N*(*t*) = *S*(*t*) + *I*(*t*) is the whole population at the time *t*.

There is a couple of equilibrium situations for the SIS model, the initial one is, *I* = 0 i.e., disease Free State, and another is,

$$\begin{array}{l} \frac{dI}{dt}=\frac{\beta SI}{N}-\gamma I=\beta I\left(1-\frac{I}{N}\right)-\gamma I=0 \end{array}$$

or,

$$\begin{array}{l} I=\left(1-\frac{\gamma }{\beta }\right)N=\left(1-\frac{1}{R_{0}}\right)N \end{array}$$

The disease spreads when $\frac{dI}{dt}>0$ or $R_{0}=\frac{\beta }{\gamma }>1$, thus the disease will transmit and attain the next stable state; else wise, it will finally attain the state of no disease.

### SIS With Vital Dynamics

Let μ and υ be the birth and death rates that are vital dynamics of the population for the SIS model. To have a stagnant population, we consider that μ = υ. Thus, the Ordinary Differential Equations for the SIS model are,

$$\begin{array}{l} \frac{dS}{dt}=\mu N-\frac{\beta SI}{N}+\gamma I-\upsilon S\\ \frac{dI}{dt}=\frac{\beta SI}{N}-\gamma I-\upsilon I \end{array}$$

Where, *N* = *S* + *I* is the whole population or *N*(*t*) = *S*(*t*) + *I*(*t*) is the whole population at the time *t*. Similarly, we can get *R*_0_ as in the case of the SIS model without vital dynamics.

### SIR and SIRS Models

**Figure 8:**
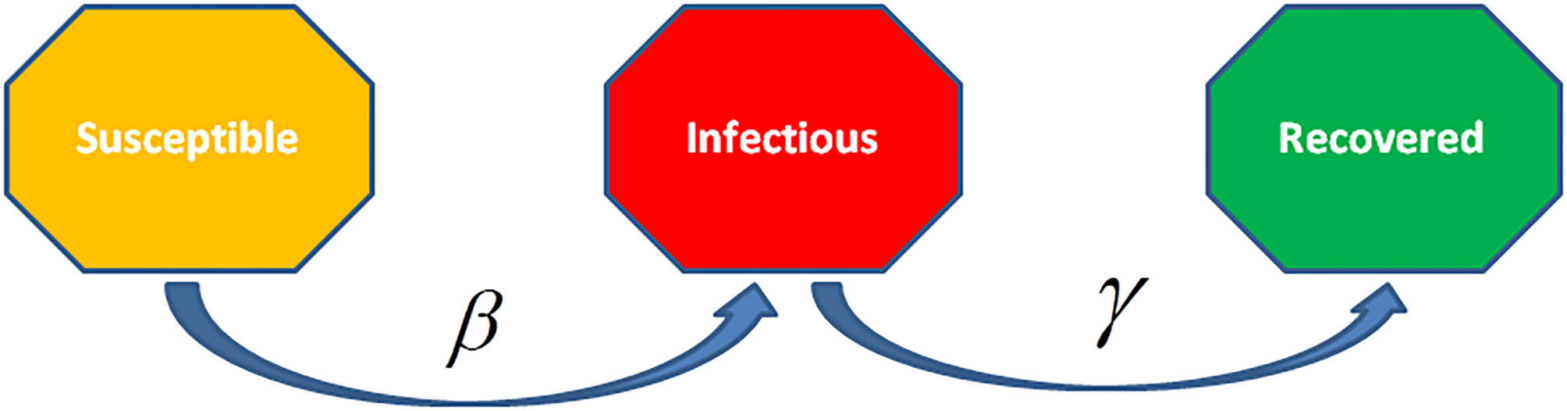
The Susceptible-Infected-Recovered (SIR) model is an epidemiological model that computes the theoretical infections with a contagious infection in a closed population over time. The family of these models involves coupled equations related to the number of susceptible people, infected cases, and recovered individuals from the disease.

The differential equations for SIR and SIRS models represent the devolution dynamics of the infectious diseases (Figure 8). In the SIR model, recovered individuals gain whole immunity to the pathogen; in the SIRS model the immunity decreases with time and persons may be infected again. The SIR/SIRS graph depicts how persons move from one fragment to another in the model. The dashed line represents how the SIR model transforms to a SIRS (Susceptible-Infectious-Recovered-Susceptible) model, lifelong immunity is not attained after recovery, and units may be re-susceptible.

The rate of infection, β, checks the spread rate which shows the chance of transmission of the disease from an infectious individual to a susceptible one. Recovery rate, $\gamma =1/\bar{d}$, is obtained through the mean time, $\bar{d}$, of infection. The SIRS model ξ represents the transmission rate from recovered to susceptible state because of decay in immunity.

$$\begin{array}{l} \frac{dS}{dt}=-\frac{\beta SI}{N}+\gamma I\\ \frac{dI}{dt}=\frac{\beta SI}{N}-\gamma I \end{array}$$

Where, *N* = *S* + *I* is the whole population or *N*(*t*) = *S*(*t*) + *I*(*t*) is the whole population at the time *t*.

### SIR Model

This model was introduced by Kermack and McKendrick (29) and has later on used to various diseases, mainly for airborne childhood diseases with lifelong immunity after recovery, like measles, mumps, rubella, and pertussis. S, I, and R in the SIR model show the number of susceptible, infected, and recovered cases, and *N* = *S* + *I* + *R* is the whole population or *N*(*t*) = *S*(*t*) + *I*(*t*) + *R*(*t*) is the whole population at the time *t*.

#### SIR Without Vital Dynamics

For the infection period with emergent eruption as compared to the lifetime of a person and the disease is non-fatal that is vital dynamics may not be considered. In such a situation, the deterministic part of the SIR model in the form of ordinary differential equations is,

$$\begin{array}{l} \frac{dS}{dt}=-\frac{\beta SI}{N}\\ \frac{dI}{dt}=\frac{\beta SI}{N}-\gamma I\\ \frac{dR}{dt}=\gamma I \end{array}$$

Where, *N* = *S* + *I* + *R* is the whole population or *N*(*t*) = *S*(*t*) + *I*(*t*) + *R*(*t*) is the whole population at the time *t*. In a population with births and deaths, an epidemic will finally vanish because of the least susceptible individuals to keep the disease. Later infected persons will not be able to start another epidemic because of the lifelong immune current population.

#### SIR With Vital Dynamics

The new births in the population with deaths and births may produce large susceptible persons to the population, having an epidemic or permitting new cases to dilation to the entire population. In a real population, the dynamics of the disease will attain a stable state. This is the situation of regional endemic for the disease.

Let μ and υ be the birth and death rates, for the SIR model with vital dynamics. For the population to be constant, we assume that μ = υ. The ordinary differential equations for the SIR model with vital parameters are,

$$\begin{array}{l} \frac{dS}{dt}=\mu N-\frac{\beta SI}{N}-\upsilon S\\ \frac{dI}{dt}=\frac{\beta SI}{N}-\gamma I-\upsilon I\\ \frac{dR}{dt}=\gamma I-\upsilon R \end{array}$$

Where, *N* = *S* + *I* + *R* is the whole population or *N*(*t*) = *S*(*t*) + *I*(*t*) + *R*(*t*) is the whole population at the time *t*.

Under the steady-state $\frac{dI}{dt}=0$.

### SIRS Model

**Figure 9:**
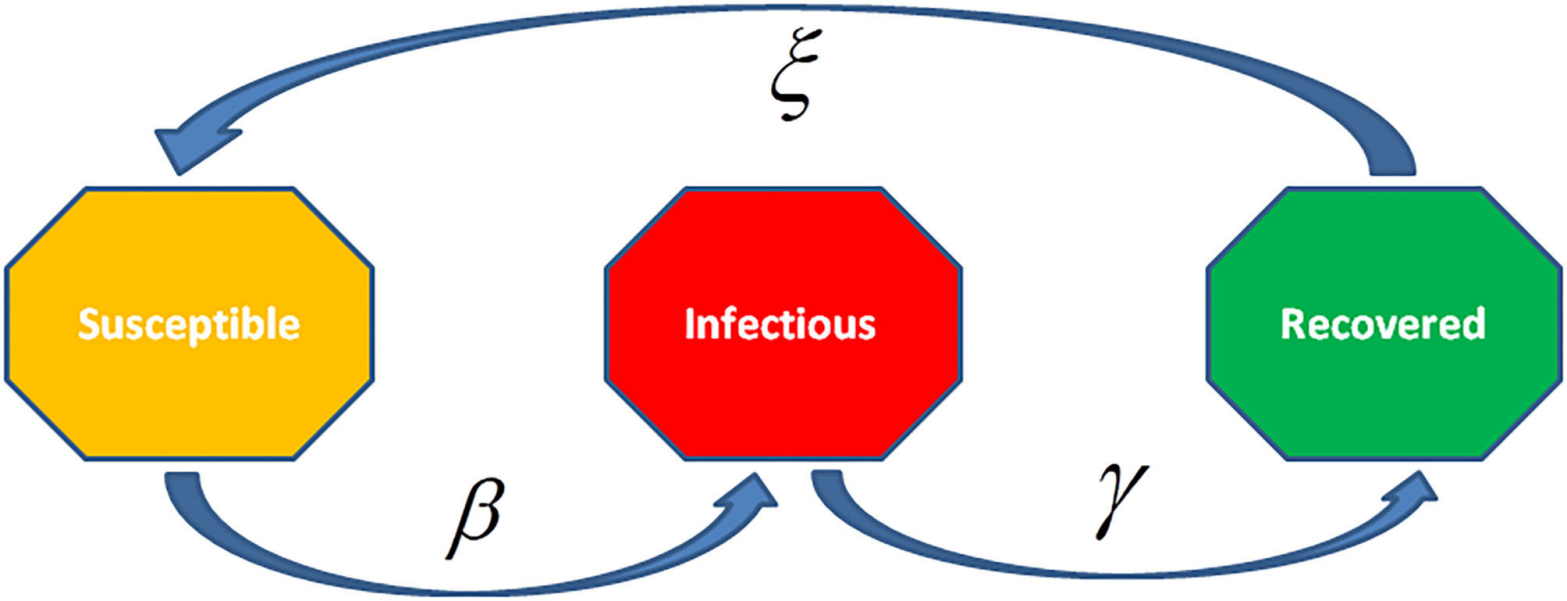
The Susceptible-Infected-Recovered- Susceptible (SIRS) model is an epidemiological model that describes the theoretically infected individuals with a contagious infection in a closed population over time. In this model, the equations are related to the susceptible, infected, and recovered number of individuals along with re-susceptible individuals for the disease.

In the SIR model, the people are lifelong immune to disease after recovery from it and this applies to various infectious diseases (Figure 9). On the other hand, there are various airborne diseases such as seasonal influenza, where an individual loses its immunity over time that is it is probable to be re-infected by the same virus. In such a case, the SIRS model is applied where recovered persons are returned to a susceptible state.

#### *SIRS Without Vital Dynamics* 

If the individuals are highly probable to belong to the susceptible population, the dynamics of the disease will be in an endemic state with damped oscillation at equilibrium. The ordinary differential equations for SIRS epidemiological model are,

$$\begin{array}{l} \frac{dS}{dt}=-\frac{\beta SI}{N}+\xi R\\ \frac{dI}{dt}=\frac{\beta SI}{N}-\gamma I\\ \frac{dR}{dt}=\gamma I-\xi R \end{array}$$

Where, *N* = *S* + *I* + *R* is the whole population or *N*(*t*) = *S*(*t*) + *I*(*t*) + *R*(*t*) is the whole population at the time *t*.

#### *SIRS With Vital Dynamics* 

The SIRS model with μ and υ as the birth and death rates can be represented in ordinary differential equations as,

$$\begin{array}{l} \frac{dS}{dt}=\mu N-\frac{\beta SI}{N}+\xi R-\upsilon S\\ \frac{dI}{dt}=\frac{\beta SI}{N}-\gamma I-\upsilon I\\ \frac{dR}{dt}=\gamma I-\xi R-\upsilon R \end{array}$$

Where, *N* = *S* + *I* + *R* is the whole population or *N*(*t*) = *S*(*t*) + *I*(*t*) + *R*(*t*) is the whole population at the time *t*. The population remains constant, if μ = υ. The steady-state for the model is $\frac{dI}{dt}=0$.

### SEIR and SEIRS Models

**Figure 10:**
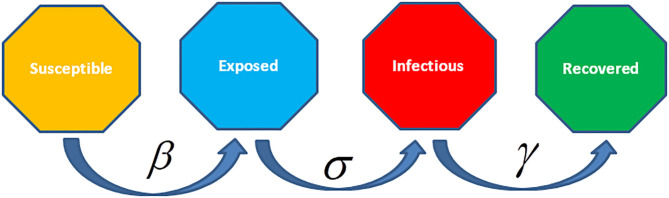
The Susceptible-Exposed-Infected-Recovered (SEIR) model is an extension of the SIR model to include an exposed but non-infectious group of individuals. This model considers the number of susceptible, exposed, infectious, and recovered individuals with no additional mortality associated with infectious disease.

In the SEIR and SEIRS models, the individuals have a long incubation period that it takes time to be exposed, so as the person is *infected* but is not still *infectious* (Figure 10). For instance, chickenpox along with vector-borne diseases, like dengue hemorrhagic fever where the pathogen is not transmitted to others by the individual. The SEIR model converses to an SEIRS (Susceptible—Exposed—Infectious—Recovered—Susceptible) model, where recovered individuals are re-susceptible. For instance, rotavirus and malaria have long incubation periods with only temporary immunity. The infectious rate, β, reduces the chance of transmission of disease from an infectious person to a susceptible one. The incubation rate, σ, represents the rate to be infected (mean time of incubation is 1/σ). Recovery rate, γ = 1/$\bar{d}$, representing the mean time, $\bar{d}$, of infection. The SEIRS model ξ is the rate by which recovered persons become susceptible because of loss of immunity.

### SEIR Model

There are diseases where the unit is infected itself but it does not infect others. This gap between becoming infected and the infectious state may be applied in the SIR model by adding a latent/exposed population, E, and allow infection but no infectious persons move from S to E and from E to I.

#### *SEIR Without Vital Dynamics* 

For a closed population with no vital dynamics, the SEIR model is:

$$\begin{array}{l} \frac{dS}{dt}=-\frac{\beta SI}{N}\\ \frac{dE}{dt}=\frac{\beta SI}{N}-\sigma E\\ \frac{dI}{dt}=\sigma E-\gamma I\\ \frac{dR}{dt}=\gamma I \end{array}$$

Where, *N* = *S* + *E* + *I* + *R* is the whole population or *N*(*t*) = *S*(*t*) + *E*(*t*) + *I*(*t*) + *R*(*t*) is the whole population at the time *t*.

As there is a delay for an individual to be infected, the secondary spread from this individual will take more time as compared to the SIR model, where there is no delay. Thus, adding a longer delay period will produce slow growth of the outbreak. However, as the model is not considering mortality, $R_{0}=\frac{\beta }{\gamma }$, will not change.

The whole outbreak of the disease is observed. Starting from rapid growth, the epidemic paralyzes the susceptible population. Finally, the virus is unable to get substantial new susceptible individuals and vanishes out. Adding the incubation time does not change the total number of infected cases.

#### *SEIR With Vital Dynamics* 

As in the SIR model with births and deaths, there is epidemic dilation since new births give more susceptible units, in a real population, the disease dynamics will tend to a stable state. For the SEIR model with μ and υ as the birth and death rates, respectively, assuming equal for maintaining a constant population, the ODE are:

$$\begin{array}{l} \frac{dS}{dt}=\mu N-\frac{\beta SI}{N}-\upsilon S\\ \frac{dE}{dt}=\frac{\beta SI}{N}-\upsilon E-\sigma E\\ \frac{dI}{dt}=\sigma E-\gamma I-\upsilon I\\ \frac{dR}{dt}=\gamma I-\upsilon R \end{array}$$

Where, *N* = *S* + *E* + *I* + *R* is the whole population or *N*(*t*) = *S*(*t*) + *E*(*t*) + *I*(*t*) + *R*(*t*) is the whole population at the time *t*.

### SEIRS Model

**Figure 11:**
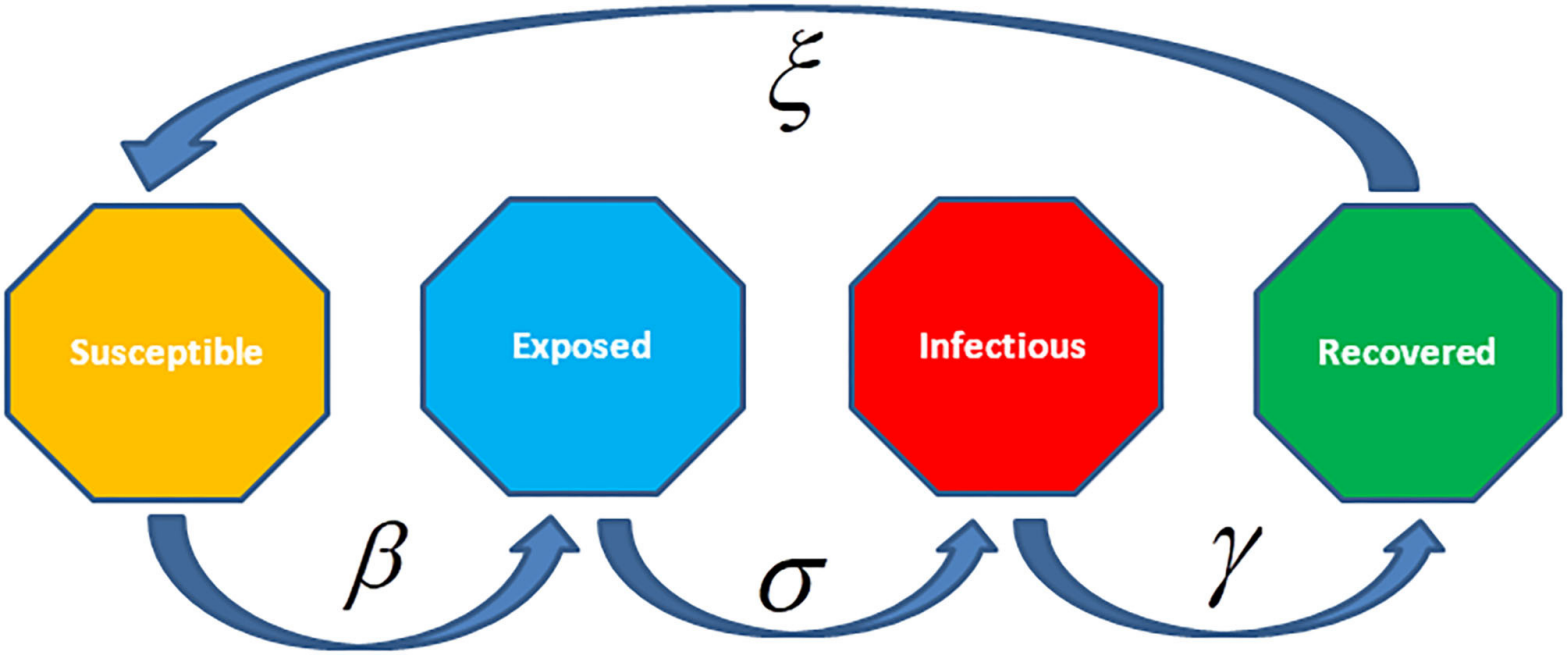
The Susceptible-Exposed-Infected-Recovered- Susceptible (SEIRS) model considers people carry lifelong immunity to disease after recovery, but for many diseases, the immunity deteriorates over time. In such cases, the SEIRS model is applied to permit recovered individuals to come back to a susceptible state. The parameter ξ represents the rate to be susceptible after recovery because of decay in immunity.

In this model, individuals have lifelong immunity for the disease after recovery, but in various diseases, the immunity goes to vanishes with time (Figure 11). Thus, SEIRS model is applied to permit the recovered persons to come back to a susceptible state. Moreover, the recovered persons turn back to the susceptible state by the rate ξ, because of decay in immunity? If the susceptible population grows sufficiently, the dynamics will be endemic with damped oscillation at equilibrium. The SEIRS model is:

$$\begin{array}{l} \frac{dS}{dt}=-\frac{\beta SI}{N}+\xi R\\ \frac{dE}{dt}=\frac{\beta SI}{N}-\sigma E\\ \frac{dI}{dt}=\sigma E-\gamma I\\ \frac{dR}{dt}=\gamma I-\xi R \end{array}$$

Where, *N* = *S* + *E* + *I* + *R* is the whole population or *N*(*t*) = *S*(*t*) + *E*(*t*) + *I*(*t*) + *R*(*t*) is the whole population at the time *t*.

#### *SEIRS With Vital Dynamics* 

The SEIRS model with vital dynamics having vital parameters μ and υ as birth and death rates, respectively, maintaining a stable population, i.e., μ = υ. In the stable state $\frac{dI}{dt}=0$, the Ordinary Differential Equations are,

$$\begin{array}{l} \frac{dS}{dt}=\mu N-\frac{\beta SI}{N}+\xi R-\upsilon S\\ \frac{dE}{dt}=\frac{\beta SI}{N}-\upsilon E-\sigma E\\ \frac{dI}{dt}=\sigma E-\gamma I-\upsilon I\\ \frac{dR}{dt}=\gamma I-\xi R-\upsilon R \end{array}$$

Where, *N* = *S* + *E* + *I* + *R* is the whole population or is the whole population at the time *t*.

## Recent Statistical Modeling Attempts for COVID-19 Disease Spread

A reasonably good body of literature is growing recently on various epidemiological models on COVID-19 disease proposed by various researchers. For instance, Hoertel et al. (44) suggested a random Agent-Based Microsimulation (ABM) model for the COVID-19 epidemic in France. They have shown the forceful effect of competing for non-pharmaceutical interventions on the accumulative incidence of the disease and mortality, and intensive care units (ICUs) -bed occupancy. They also discussed the model with different suppressive measures and suggested the lockdown period. They have calculated *R*_0_ for their model to predict the pattern of the infection spread. They have described that their model depends on current knowledge and updated presupposition, as in the case of all modeling work happens, in their work, they have additionally shown using empirical data that lockdown and physical distancing and mask-wearing are emphatic in downing the epidemic and decreasing mortality rates but are not sufficient to inhibit Inevitable ICUs admissions and a second lockdown.

De-Souza et al. (45) reported and contextualized epidemiological, demographic, and clinical results for COVID-19 cases throughout the first three months of the pandemic. They derived through their model that the value of *R*_0_ was 3.1 with 95% Bayesian credible interval as [2.4–5.5] with a greater median but overlapping credible intervals as compared to some other gravely affected countries. They have also shown a positive correlation between higher per capita income sub-population and COVID-19 diagnosis while severe acute respiratory infection cases with unknown etiology were associated with lower per-capita income sub-population in Brazil. Hao et al. (46) suggested SAPHIRE model to reconstruct the full-spectrum dynamics of COVID-19 in Wuhan, China which is an extension of the classical SEIR model, which is also discussed erstwhile in this review. They have devised the new model by adding demographic and clinical components of the model. They have calculated the lower bounds of the infection along with *R*_0_ and shown than the value of *R*_0_ was 3.54 for their model with 95% credible interval as (0.23–0.33) and predicted the projection of the pandemic through their model that it is going to finish 96% till 8 March 2020. Gaglione et al. (47) worked on an epidemiological model to estimate of parameters of infection and recovery, and to follow and predict the epidemiological graph with reasonable accuracy applying to real data from the Lombardia region in Italy, and from the USA. They also did Bayesian sequential estimation of the parameters under consideration with a specific prior distribution. They also demonstrated the prediction of the pandemic spread by their dynamic and observation model along with the basic reproduction number *R*_0_. Lavezzo et al. (48) utilized the prevalence estimates of Vo' at the primary and secondary surveys to calibrate an improved susceptible–exposed–infectious–recovered compartmental model of SARS-CoV-2 transmission that incorporates symptomatic, presymptomatic and asymptomatic infections, virus detectability before and after the infectious duration and the effect of the lockdown. They assumed that presymptomatic, symptomatic, and asymptomatic infections transmit the virus. They calculated the value of *R*_0_ for their model and they also performed the Mann-Whitney *U*-test, a non-parametric test to test the equality of two independent means of the first and second survey populations along with some other statistical tests as well. Flaxman et al. (49) have studied the impact of main interventions across 11 European countries from the beginning of the COVID-19 epidemics in February 2020 until 4 May 2020, when lockdowns lifting was to be started. They shown drawbacks through their model, from the given deaths to estimate transmission that took place many weeks previously, allow time lag between infection and death. They used a partial pooling of information between countries, with both individual and shared impacts on the time-varying reproduction number (*R*_*t*_). Their model believes on the fixed estimates of some epidemiological parameters (such as the infection fatality rate), not including importation or subnational variation and considers that changes in *R*_*t*_ has quick response to interventions rather than slow changes in behavior. They estimated that for all of the countries of study current interventions are sufficient to drive *R*_*t*_ below 1 (probability *R*_*t*_ < 1.0 is >99%) and get control over epidemic. Malavika et al. (50) utilized the Logistic growth model for short term forecasting; SIR models to predict the optimum number of active cases and peak time and Time Interrupted Regression model to evaluate the effect of lock-down and other interventions.

Other authors used different statistical techniques to analyze and predict the transmission dynamics of the COVID-19 pandemic. Baggett et al. (51) utilized descriptive statistics for characterization of the sample under consideration, the percentage of positive PCR test results, and the symptom profile of persons with PCR-confirmed infections of COVID-19. Partners Health Care Human Research Committee exempted their study with a waiver of informed consent. The barricades of this study were cross-sectional nature at only one shelter in Boston where numerous symptomatic persons had been eliminated by prior symptom screening or self-referrals and not to care. These results advocate PCR testing of asymptomatic shelter residents if a symptomatic person with COVID-19 is identified in the same shelter. Hsiang et al. (95) study different statistical model fitting and epidemiological studies for the transmission dynamics of COVID-19 disease. They compiled the data on 1,700 local, regional and national non-pharmaceutical interventions which were assigned during the current pandemic across different localities in China, South Korea, Italy, Iran, France, and the United States. Then they applied reduced-form econometric methods, generally applied to see the impact of policies on economic growth to empirically measure the effect that these anti-contagion policies have had on the growth rate of infections. They analyzed that anti-contagion policies have remarkably and virtually made slow this growth and can be helpful in making well-informed decisions regarding whether or when these policies should be applied, intensified, or lifted, and they may support policy-making in the more than 180 other countries in which COVID-19 has been reported.

Banerjee et al. (52) presented the population-based cohort data on the primary and secondary care electronic health records from England. They have shown the prevalence of underlying conditions defined by Public Health England guidelines in persons with age 30 years and above registered with a practice between 1997 and 2017, utilizing valid and freely available phenotypes for each situation. They have estimated 1-year mortality in every situation, through simple models and calculated excess COVID-19-related deaths, considering relative effect as relative risks of the COVID-19 pandemic. They also made an online, public, prototype risk calculator for estimating excess deaths. Several other India specific forecasting daily and the cumulative number of cases of infections of COVID-19 have been published recently using modeling (42, 53–55). Similarly, Rai et al. (56) have described the impact of social media advertisements on the transmission dynamics of COVID-19 pandemic in India.

## Conclusion

In this review, we have discussed the different types of statistical modeling used for predicting infectious disease spread. The first one among them is distribution fitting, wherein most of the infectious diseases as a large number of cases are infected so the Gaussian or the normal distribution is fitted to the observed data and the parameters of the distribution are estimated based on the sample observations and the peak and the 99 percent downfall, etc. are calculated using the area property of normal distribution through these estimates of the parameters of the fitted distribution. In a recent report by Hamzaha et al. (57). The worldwide data on the COVID-19 outbreak was analyzed and the fate of the disease was predicted using distribution techniques.

The second type of infectious disease modeling has been discussed using the epidemiological models which include SI, SIS, SIR, SIRS, SEIR, and SEIRS models. It has also been discussed which model should be used for specific infectious diseases and why of the basis of its nature. These epidemiological models *R*_0_ and the average reproduction number by the infected person, *R* have been discussed and the conditions for these numbers have been discussed for the disease to be controlled or finished. These models have been discussed with the suppressive strategies and without suppressive strategies along with and without the vital parameters. Similarly, UCLA Statistical Machine Learning Lab (2020) reported epidemiological models for COVID-19 through modified SEIR models for predicting the dynamics among the cumulative confirmed cases and mortality rates of COVID-19. Li et al. (36) envisaged Mathematical Modeling and forecasting of COVID-19 spread and its importance to epidemic stopover and curb measures. Jewell et al. (37) described forecasting mathematical models of the COVID-19 pandemic their principles and value of projections. Ndaïrou et al. (38) discussed Mathematical modeling of COVID-19 spread dynamics through a case study of Wuhan. Griffiths (39) observed and discussed whether mathematical modeling can solve the current COVID-19 crisis. Kucharski et al. (40) worked on anon dynamics of spread and control of COVID-19 through a mathematical modeling study.

The third type of infectious disease modeling has been discussed using the time series modeling. In this time series disease modeling, different types of time series models for different infectious diseases have been discussed. These time series models include AR, MA, ARMA, ARIMA, and SARIMA models. These models have been estimated for forecasting using well-known Box-Jenkins method. In recent studies, the authors have carried out the prediction and analysis of COVID-19 through the time series models. They attempted to fit three models namely logistic, Gompertz and Bertalanffy models to the COVID-19 data set and showed that the logistic model is best among the three models used for prediction in this study. Papastefanopoulos et al. (58) analyzed COVID-19 by comparing different time series models to predict the percentage of active cases in a population. Yonar et al. (59) described the Modeling and prediction for COVID-19 pandemic cases using the curve estimation models, the Box-Jenkins, and exponential smoothing methods for the chosen countries of G8 countries, Germany, United Kingdom, France, Italy, Russian, Canada, Japan, and Turkey between 1/22/2020 and 3/22/2020 through ARIMA model.

## Further Suggestions and Future Prospectives

The three types of infectious disease modeling work very well but still, there is room for improvements in these modeling methods. Following are some suggestions for improving these modeling methods, respectively.

- As far as the fitting of the Gaussian distribution is concerned, there must be an appropriate sample size to get the best estimates for forecasting the features of the fitted distribution. If the sample size is not appropriate as per the population size, the estimates will not be best that is why we have seen in the case of COVID-19 many times the 95 and 97% confidence interval of the disease time to be finished are revised. Additionally, there must be only one distribution fitting as some authors have fitted different distribution for different stages and they have presented the problem in different stages, which may not be a true depiction.
- In the case of epidemiological modeling, the model prediction will be accurate if some of the constraints or the suppressive measure which are qualitative in nature must be considered as the explanatory variable and it should not be only a dummy variable rather these should be taken as fuzzy variables. One more thing for modeling the data may be true and complete for which there must be maximum testing as there are cases that are asymptomatic and they are prone to spread the disease. Further, Benford's law (60) and Zipf's law (61) must be applied to epidemiological models to establish the guidelines for reporting fraud free epidemiological data.
- In the case of time series modeling again the problem of appropriate sample size remains the same to get the best estimates of the parameter of the model under consideration. Many variables are not included in the model but they are having an impact on the output so they must be included to get a more accurate prediction.
- The true representation of the data is the soul of the infectious disease modeling. Consequently, it is of prime importance for the modeling and prediction of the phenomenon accurately, that the data should be true and complete. In most of the cases the available data is incomplete and not truly represent the population and in such situation modeling efforts miserably fail to predict accurately. This is very true in the case of COVID-19, which is changing its potential of transmission very dynamically. There could be an n-number of factors, which may initiate an outbreak and intensify the spread of the disease. Therefore, as many as possible, such factors, which are quantifiable, must be included in the model for achieving a robust and accurate prediction.

## Author Contributions

SKY and YA have collected the literature, analyzed the data, and written the manuscript draft. Both authors contributed to the article and approved the submitted version.

## Conflict of Interest

The authors declare that the research was conducted in the absence of any commercial or financial relationships that could be construed as a potential conflict of interest.
